# Evaluating the Application of the RE-AIM Planning and Evaluation Framework: An Updated Systematic Review and Exploration of Pragmatic Application

**DOI:** 10.3389/fpubh.2021.755738

**Published:** 2022-01-26

**Authors:** Danielle D'Lima, Tayana Soukup, Louise Hull

**Affiliations:** ^1^Department of Clinical, Educational and Health Psychology, Centre for Behaviour Change, University College London, London, United Kingdom; ^2^Centre for Implementation Science, Health Service and Population Research Department, King's College London, London, United Kingdom

**Keywords:** RE-AIM framework, planning frameworks, evaluation frameworks, implementation frameworks, implementation models, implementation theories, systematic review

## Abstract

**Background:**

RE-AIM is one of the most widely applied frameworks to plan and evaluate the implementation of public health and health behavior change interventions. The objective of this review is to provide an updated synthesis of use of the RE-AIM (Reach Effectiveness Adoption Implementation and Maintenance) planning and evaluation framework and explore pragmatic use (i.e., partial application of the framework) and how this is reported.

**Methods:**

Systematic review. MEDLINE (R) and PsycINFO were searched, via the Ovid interface, between January 2011 and December 2017. Studies that applied RE-AIM as a planning and/or evaluation framework were included.

**Results:**

One hundred fifty-seven articles met inclusion criteria. One hundred forty-nine reported using RE-AIM for evaluation, three for planning and five for planning and evaluation. Reach was the most frequently reported dimension (92.9%), followed by implementation (90.3%), adoption (89.7%), effectiveness (84.5%), and maintenance (77.4%). One hundred forty-seven/one hundred fifty-seven articles originated from high-income economy countries. Within a sub-set analysis (10% of included articles), 9/15 articles evaluated all dimensions. Of the 6/15 articles that did not evaluate all dimensions, five provided no justification for pragmatic application.

**Conclusions:**

RE-AIM has gained increased use in recent years and there is evidence that it is being applied pragmatically. However, the rationale for pragmatic use is often not reported.

**Systematic Review Registration:**

PROSPERO (CRD42017054616).

## Introduction

There is growing awareness of the importance of using theories, frameworks and models (TFMs) in implementation research and practice (1–3). TFMs summarize the current state of scientific knowledge, help to structure thinking, facilitate the accumulation of evidence, and offer a common language, supporting more effective communication across key implementation stakeholders (4–6). In practice, TMFs also offer an understanding of the factors affecting implementation success and failure and support more effective intervention development and evaluation (3, 4, 6).

For TMFs to be used optimally, researchers and practitioners need to identify, select and apply them appropriately (7–9). Furthermore, evaluation and refinement of TMFs are dependent on clear reporting of which TMFs have been selected and why, how they have been applied, for what purpose, and with what outcome (10).

One of the most widely cited and used frameworks to plan and evaluate the implementation of public health and health behavior change interventions is the RE-AIM planning and evaluation framework (11, 12). It was originally developed to increase the reporting of internal and external validity factors for public health interventions (12, 13).

The RE-AIM framework consists of five domains, reach, effectiveness, adoption, implementation and maintenance, which can be considered at both the planning and evaluation stages of implementation. The authors of the RE-AIM framework have created a website to support researchers and practitioners in understanding and applying the framework (http://www.re-aim.org/). Definitions for each of the domains (as cited on the RE-AIM website) are included below:

- Reach is defined as “The absolute number, proportion, and representativeness of individuals who are willing to participate in a given initiative, intervention, or program, and reasons why or why not.”
- Effectiveness is defined as “The impact of an intervention on important outcomes. This includes potential negative effects, quality of life, and economic outcomes. Also important to understand variability across subgroups (heterogeneity) and why.”
- Adoption is defined as “The absolute number, proportion, and representativeness of settings and staff who are willing to initiate a program or approve a policy, and reasons why or why not. Note settings and staff can each be multi-level: delivery staff nested under supervisors, clinics or schools, health systems, communities, etc.”
- Implementation is defined as “At the setting level, implementation refers to how closely staff members follow the program that the developers provide. Importantly, this includes consistency of delivery as intended, adaptations made to the intervention or implementation strategies, and the time and cost of the program.”
- Maintenance is defined as “At the setting level, the extent to which a program or policy becomes part of the routine organizational practices and policies. Newer guidance includes tailoring the time frame of maintenance to specific issues and programs, and evaluation of adaptations made for sustainment. At the individual level, maintenance refers to the longer-term effects of a program on outcomes after the most recent intervention contact. Time frame of maintenance assessment should be tailored to the program and health issue.”

Since its development in 1999 (12) RE-AIM has been used to guide planning and evaluation of the implementation of multiple interventions across a variety of content areas, settings, and populations (14). It has also been applied retrospectively in systematic reviews of intervention studies (15–20). In 2013, Gaglio et al. published a systematic review of the use of the RE-AIM framework over time, and reported that the framework has been applied broadly, that few studies report on all five dimensions or all evaluation criteria within a RE-AIM dimension, and identified common problems in application across all domains (14).

In recent years, the RE-AIM dimensions have evolved with several refinements and extensions including updated recommendations for use (21). RE-AIM was originally positioned as a quantitative *post-hoc* evaluation framework (12). However, it is now widely regarded as both a planning and evaluation framework with qualitative and mixed-methods applications strongly encouraged (13, 22). More recently, its developers have argued that RE-AIM can be used in a more iterative manner to inform and guide adaptations to interventions and implementation strategies during the implementation process (23). A recent publication also details an extension of RE-AIM to enhance evaluation of the sustainability of evidence-based programs, policies, and practices (EBIs) (24).

The challenges of applying the RE-AIM framework in its entirety have also been highlighted and discussed (13, 25). Its developers recognize that assessment of all RE-AIM dimensions may not be feasible, especially outside the context of research projects with substantial funding (13). Hence, the developers have suggested that more pragmatic applications of RE-AIM (i.e., partial application of the framework) may be warranted (13). Fully applying RE-AIM may not be necessary or appropriate for all studies, with the developers encouraging users to identify and assess the RE-AIM dimensions that are ‘most valued and appropriate for their particular question, setting, stakeholders, and stage of research' (13). This focus is supported by ‘simplified, pragmatic, user-centered, and stakeholder-centered' recommendations to increase RE-AIM use (26).

Such discussions on pragmatic use are not limited to RE-AIM and there is growing evidence that implementation TFMs are not always applied in their entirety (14, 27–29). The reasoning behind pragmatic use needs to be clearly articulated to demonstrate that it is justified (26), and to support evaluation and refinement of TMFs (including RE-AIM) over time.

We set out to provide an updated synthesis of RE-AIM use over time [update of review by Gaglio et al. (14)] and explore the pragmatic application of the framework and how this is reported. We define pragmatic application as the partial (i.e., not in its full form) application of the RE-AIM framework. This definition, although simplistic, provides an objective way to assess the pragmatic application of RE-AIM and is in line with the pragmatic application of the framework recently described by one of the original developers (26).

The current systematic review has four objectives:

1. To document the evolution of RE-AIM application by providing an updated synthesis of RE-AIM use from 2011 to 2017.
2. To compare the results to a systematic review of RE-AIM use over time from 1999 to 2010, published by Gaglio et al. (14).
3. To provide an in-depth exploration of the pragmatic use of RE-AIM at a (1) dimension level (e.g., reach) and (2) evaluation criteria level (e.g., exclusion criteria (% excluded or characteristics), in a sub-set of articles meeting inclusion criteria.
4. To provide an in-depth exploration of the reasoning and justification for full and pragmatic use of RE-AIM, at a dimension level, in a sub-set of articles meeting inclusion criteria and document the challenges and benefits of applying RE-AIM reported by authors.

## Methods

### Study Design

We conducted a systematic literature review. The Preferred Reporting Items for Systematic Reviews and Meta-Analyses (PRISMA) statement and checklist were adhered to.

### Systematic Review Protocol

The review protocol was registered with the International Prospective Register of Systematic Reviews (PROSPERO), registration number: CRD42017054616 and deviations from the protocol are reported below and in Supplementary File 1.

Several protocol deviations were made to reflect more recent developments in understanding and evaluating the application of RE-AIM, and the changing capacity of our research team (the number of researchers contributing to the review decreased from five to three).

Below we detail our revised data extraction process and all protocol deviations are listed in Supplementary File 1.

### Data Sources

MEDLINE (R) and PsycINFO were searched, via the Ovid interface, for relevant articles. The following limits were applied: English Language and 01/01/2011-31/12/2017. The last search was performed on the 04/01/2018.

### Search Strategy

The search term “RE-AIM” was used to search for relevant articles.

### Inclusion and Exclusion Criteria

We applied the following inclusion and exclusion criteria:

#### Inclusion

1. Articles were included if they reported the use of any of the five RE-AIM dimensions (reach, effectiveness, adoption, implementation, and maintenance)
2. Reported in English
3. Published on or after 1st January 2011 to 31st December 2017.

#### Exclusion

1. Articles were excluded if they were commentaries, theoretical papers, published abstracts, dissertations, book chapters, editorials, or did not report on the use of RE-AIM for planning or evaluation of a study, program, or policy.

Studies were not excluded based on methodology. However, systematic reviews that applied RE-AIM to collate data across multiple primary studies (that had not used RE-AIM for planning or evaluation of a study, program, or policy) were excluded.

### Data Screening

Two researchers (DD & LH) independently reviewed all titles and abstracts to identify relevant articles. Following title and abstract screening, DD & LH independently reviewed all articles at full-text stage to identify articles that met inclusion criteria. Throughout this process, any discrepancies were identified and resolved by discussion until consensus was reached.

### Data Extraction

In line with our original review protocol, we developed and piloted a standardized data extraction form (see Supplementary File 2). This form was based on the data presented in the systematic review reported by Gaglio et al. (14). However, we experienced significant challenges as the associated evaluation criteria, relating to each RE-AIM dimension, were not explicitly operationalised in the Gaglio et al. review. Deviations in how authors operationalised the evaluation criteria level varied considerably making it difficult to extract data consistently and objectively across all included articles. This difficulty highlighted the need for multiple deviations to the original review protocol, and stages to our data extraction process, to ensure data were extracted objectively and consistently, to account for recent developments in the RE-AIM literature (13). The final data extraction process is reported below and mapped to the four objectives of the review.

#### Stage One

The first objective of the review was to provide an updated synthesis of RE-AIM use over time, 2011–2017. The second objective of the review was to compare the results to a systematic review of RE-AIM use over time, 1999–2010, published by Gaglio et al. (14), and document the evolution of RE-AIM application. (See Supplementary File 2), section *Criteria Relating to the Key Characteristics of the Article* for details of data extracted from all articles that met our inclusion criteria.

Data were extracted by four individuals (DD, TS, RD, ZK). Data extraction for all articles was checked for accuracy by LH and DD. Where discrepancies were identified, LH and DD discussed them until consensus was reached.

#### Stage Two

The third objective of the review was to provide an in-depth exploration of the pragmatic use of RE-AIM, in a sub-set of articles meeting inclusion criteria. We applied the full data extraction form (see Supplementary File 2, section *Criteria Relating to Reporting at the RE-AIM Dimension Criteria Level*) to a random selection of 10% (*n* = 15) of the included articles. The decision to conduct the sub-analysis on 10% of articles was made in light of the depth of data extraction and the capacity of our research team. We used an online random number generator to select articles to include in the sub-set analysis (https://www.calculator.net/random-number-generator.html). Data from each article were extracted by one reviewer (either DD or LH) and then checked by the other reviewer (either DD or LH) with the objective to extract as much relevant data for each evaluation criteria as possible including any contextual information on ambiguities about how it had been defined and/or reported. Discrepancies in extraction were reviewed and resolved through discussion and any key themes in ambiguities were documented.

#### Stage Three

The fourth objective of the review was to provide an in-depth exploration of the reasoning and justification for full and pragmatic use of RE-AIM, in a sub-set of articles meeting inclusion criteria. In line with recent developments in the RE-AIM literature (13, 26), it is important to acknowledge that pragmatic application of selected RE-AIM dimensions is encouraged by RE-AIM developers (13, 14), as long as authors explicitly document and justify their decisions about what will and will not be used. Finally, we extracted data from the sub-set of articles included at stage two on the following two items: justification for evaluating and/or not evaluating RE-AIM dimension(s); and challenges and benefits of applying RE-AIM, as reported and described by authors (see Supplementary File 2, section *Criteria Relating to Pragmatic Application of RE-AIM*).

## Results

**Figure 1:**
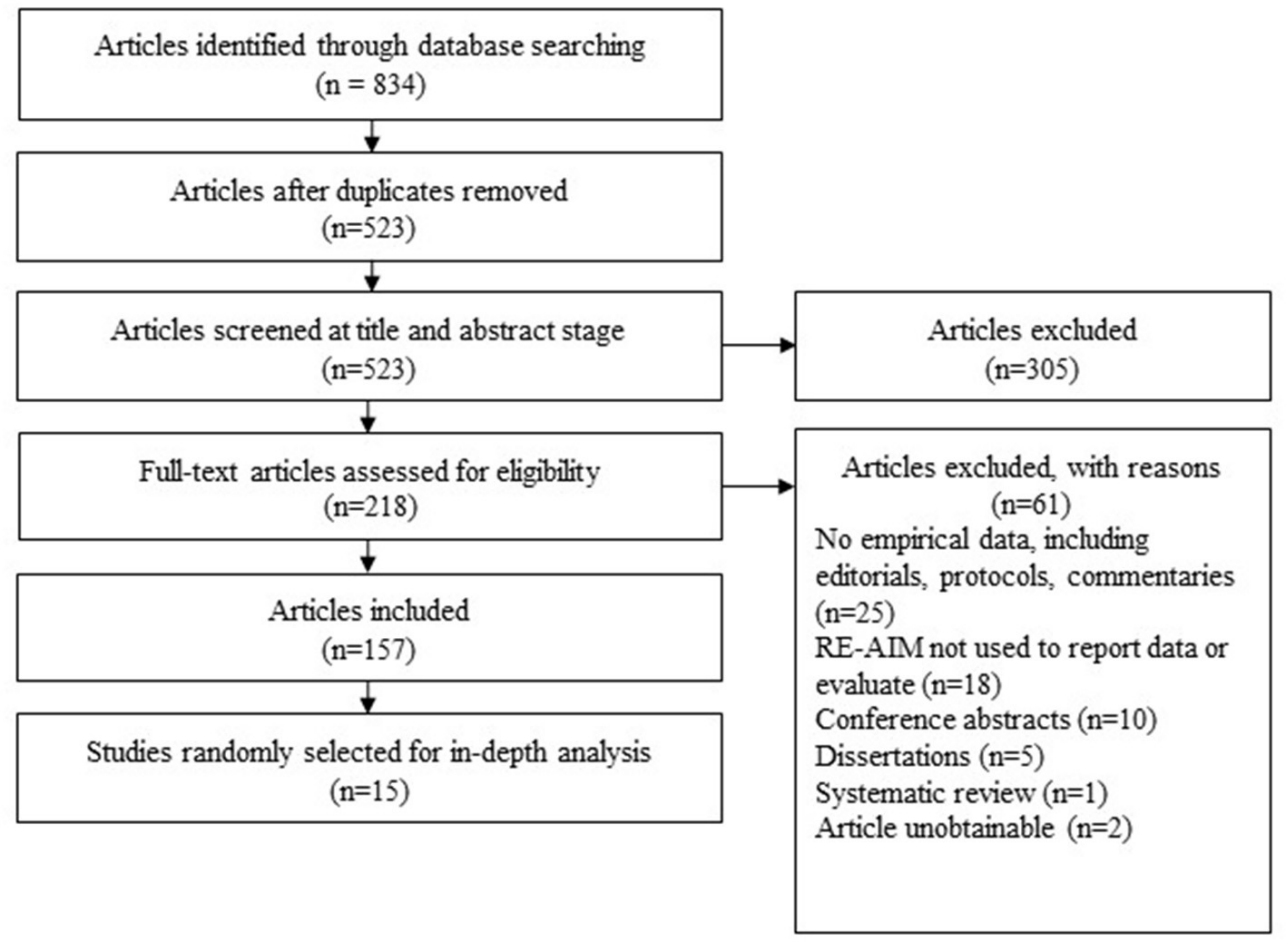
PRISMA flowchart of results.

The search retrieved 834 articles. After removing duplicates, 523 articles remained for screening at the title and abstract stage, and 305 were subsequently excluded. The remaining 218 articles were reviewed at full-text stage with 157 meeting inclusion criteria. Of the 157 included articles, 15 (approximately 10%) were randomly selected for the sub-analyses. (See Figure 1) for PRISMA flowchart.

We present the results of the review according to the previously described review stages and associated objectives.

### Stage One

#### Updated Synthesis of RE-AIM Use Over Time (2011–2017)

A high-level summary, including topic area, nature of RE-AIM application and RE-AIM dimensions evaluated, of all included articles (n=157) can be found in Supplementary File 3.

##### Nature of RE-AIM Use

RE-AIM was used as an evaluation framework in 149/157 articles, as a planning framework in 3/157, and as a planning and evaluation framework in 5/157.

##### RE-AIM Dimension Use

Of the included articles that explicitly reported using RE-AIM to plan and/or evaluate implementation at the dimension level (*n* = 155/157), 107 (69.0%) reported all five RE-AIM dimensions, 21 (13.5%) reported four, 10 (6.5%) reported three, eight (5.2%) reported two, and nine (5.8%) reported one. Fourteen different RE-AIM dimension combinations (i.e., which of the five dimensions were evaluated) were reported. Reach was the most frequently reported RE-AIM dimension (92.9%) followed by implementation (90.3%), adoption, either at the setting and/or staff level (89.7%), effectiveness (84.5%) and maintenance, either at the setting and/or individual level (77.4%). For a comparison of the percentage and difference across the two reviews, (see Table 1).

**Table 1:** Percentage of articles reporting on RE-AIM dimensions and combinations, across the two reviews.

**RE-AIM dimensions reported**	**Frequency percentage reported**		**Frequency percentage difference between reviews**
	**Gaglio et al. (14) (*n* = 71)**	Current review (*n* = 155^*^/157)	
1 dimension	5.6%	5.8%	0.2%↑
2 dimensions	6.4%	5.2%	1.2%↓
3 dimensions	9.9%	6.5%	3.4%↓
4 dimensions	15.5%	13.5%	2.0%↓
5 dimensions	62.0%	69.0%	7.0%↑
Number of combinations	14	14	No difference
Reach	91.5%	92.9%	1.4%↑
Effectiveness	77.5%	84.5%	7.0%↑
Adoption (setting and/or staff level)	75.3%	89.7%	14.4%↑
Implementation	90.1%	90.3%	0.2%↑
Maintenance (setting and/or individual level)	71.8%	77.4%	5.6%↑

* *Analysis based on 155/157 articles as although all articles applied RE-AIM, explicit reference to planning and/or evaluation, at dimension level, was not reported by two authors. ↑ indicates that the percentage of articles reporting on RE-AIM dimensions and combinations across the two reviews (Gaglio et al's and our own) has increased. ↓ indicates that the percentage of articles reporting on RE-AIM dimensions and combinations across the two reviews (Gaglio et al's and our own) has decreased*.

##### Journals Most Frequently Publishing Articles Using RE-AIM

The five journals that most frequently published RE-AIM articles were BMC Public Health (13 articles), Translational Behavioral Medicine (*n* = 12), BMC Implementation Science (*n* = 8), Health Promotion Practice (*n* = 6), and Canadian Journal of Diabetes (*n* = 4). In comparison, Gaglio et al. (14) reported the five journals that most frequently published RE-AIM articles were American Journal of Preventive Medicine (*n* = 7), Annals of Behavioral Medicine (*n* = 4), American Journal of Public Health (*n* = 3), and Patient Education Counseling (*n* = 3).

##### Year of Publication

This review includes 157 articles published over a 7-year period, while the review conducted by Gaglio et al. (14) includes 71 articles published over an 11-year period. This comparison represents an upward trend in the articles reporting the use of RE-AIM over time with a mean yearly publication rate of 6.5 articles, between 1999 and 2010, increasing to 22.4 articles, between 2011 and 2017.

##### Study Design

RE-AIM dimensions were evaluated using quantitative methods in 61/157 articles, qualitative methods in 20/157, and mixed methods in 76/157 articles.

##### Fequency of RE-AIM Use by Country

**Figure 2:**
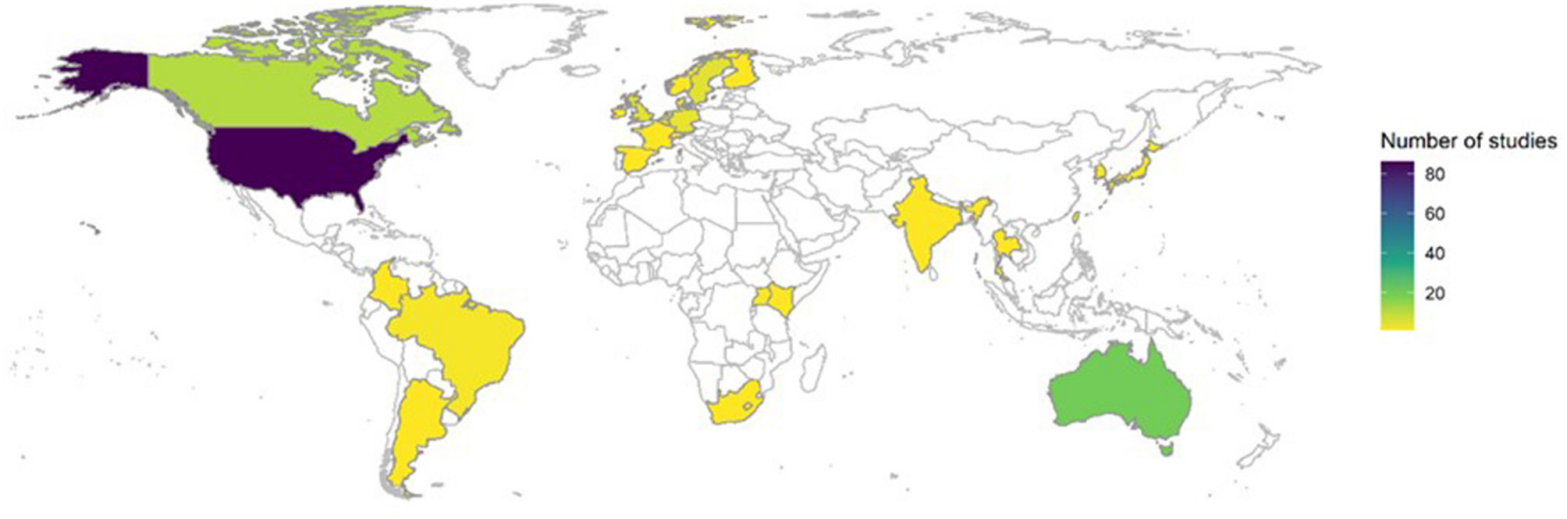
Frequency of RE-AIM use by country.

Articles originated from 26 countries. The five countries producing the most articles were the USA (*n* = 86), Australia (*n* = 18), Canada (*n* = 11), the Netherlands (*n* = 6), Sweden (*n* = 5), and the UK (*n* = 5). Three articles were international studies. The number of articles produced from each country is presented in Supplementary File 4. One hundred forty-seven/one hundred fifty-seven articles originated from high-income economy countries, 6/157 from upper-middle income economy countries, 3/157 from lower-middle income economy countries, and 1/157 from lower-income economy countries. A full breakdown of the number of articles across countries and income classification groups (low, lower-middle, upper-middle and high), and a heatmap detailing the frequency of RE-AIM application across countries can be found in Supplementary File 4 and Figure 2 respectively. The income classification of a country was based on the 2018 World Bank classification criteria (30).

### Stage Two

#### In-depth Analysis of RE-AIM Application at a Dimension and Evaluation Criteria Level

Here, we report the results of an in-depth sub-analysis of RE-AIM application of 15/157 (approximately 10%) randomly selected articles. All the articles used RE-AIM as an evaluation framework. Ten studies employed mixed-methods, three used quantitative methods, and two used qualitative methods. The studies covered content areas such as physical activity, palliative care, healthy eating and smoking cessation. One to five RE-AIM dimensions were evaluated. Significant variation in the number of evaluation criteria reported was found across articles, as well as significant variation in the frequency at which individual evaluation criteria were reported. Table 2 presents the topic area, nature of RE-AIM application, study design, dimensions evaluated, and RE-AIM dimension combination (i.e., which of the 5 dimensions were evaluated) across the 15 articles. Table 3 provides an overview of the in-depth analysis for the 15 articles. For each of the five RE-AIM dimensions, we report the number of articles that evaluate each dimension and evaluation criteria. We also report additional information relating to how evaluation criteria have been defined/operationalised and/or reported for individual articles.

**Table 2:** Summary of 15 articles included for in-depth analysis of RE-AIM application.

**References**	**Topic area**	**Nature of RE-AIM application**	**Study design**	**Reach evaluated**	**Effectiveness evaluated**	**Adoption evaluated**	**Implementation evaluated**	**Maintenance evaluated**	**RE-AIM combination (number of dimensions accessed)**
Aittasalo et al. (31)	Physical activity	Evaluation	Mixed-Methods	Yes	Yes	Yes	Yes	Yes	R-E-A-I-M (5)
Anderson et al. (32)	Palliative care	Evaluation	Mixed-Methods	Yes	Yes	Yes	Yes	Yes	R-E-A-I-M (5)
Austin et al. (33)	Physical activity	Evaluation	Mixed-Methods	Yes	No	Yes	Yes	Yes	R-A-I-M (4)
Casey et al. (34)	Physical activity	Evaluation	Mixed-Methods	Yes	No	Yes	Yes	No	R-A-I (3)
Duffy et al. (35)	Smoking cessation	Evaluation	Mixed-Methods	Yes	No	Yes	Yes	Yes	R-A-I-M (4)
Folta et al. (36)	Cardiovascular disease	Evaluation	Mixed-Methods	Yes	Yes	Yes	Yes	Yes	R-E-A-I-M (5)
Folta et al. (37)	Cardiovascular disease	Evaluation	Mixed-Methods	Yes	Yes	Yes	Yes	Yes	R-E-A-I-M (5)
Jenkinson et al. (38)	Physical activity	Evaluation	Mixed-Methods	Yes	Yes	Yes	Yes	Yes	R-E-A-I-M (5)
Lee et al. (39)	Physical activity and fruit and vegetable consumption	Evaluation	Quantitative design	Yes	Yes	Yes	Yes	Yes	R-E-A-I-M (5)
Martinez-Donate et al. (40)	Healthy eating	Evaluation	Quantitative design	Yes	Yes	Yes	Yes	Yes	R-E-A-I-M (5)
Parahoo et al. (41)	Prostate cancer	Evaluation	Qualitative design	No	No	No	Yes	No	I (1)
Quinn et al. (42)	Healthy eating	Evaluation	Qualitative design	Yes	Yes	Yes	No	No	R-E-A (3)
Ulbricht et al. (43)	Tobacco smoke exposure in children	Evaluation	Quantitative design	Yes	Yes	No	No	No	R-E (2)
Van Acker et al. (44)	Physical activity	Evaluation	Mixed-Methods	Yes	Yes	Yes	Yes	Yes	R-E-A-I-M (5)
Wallace et al. (45)	Diabetes	Evaluation	Mixed-Methods	Yes	Yes	Yes	Yes	Yes	R-E-A-I-M (5)

**Table 3:** RE-AIM dimensions and evaluation criteria reported across articles included in the sub-analysis.

	**Percentage of articles reporting RE-AIM dimension and evaluation criteria**	**Pertinent findings from the current review**
**Reach**		
Reach evaluated	93.3% (31–40, 42–45)	
Exclusion Criteria (% excluded or characteristics)	0.0%	Two articles reported data that would allow the percentage to be calculated (35, 43)
Percentage of individuals, who participate, based on valid denominator	46.7% (31, 35–37, 43–45)	One article did not report the percentage based on a valid denominator but reported data that would allow readers to calculate the percentage (38) One article reported on the percentage of individuals, who participate, based on a valid denominator at the school (i.e., organizational level) rather than individual level (33)
Characteristics of participants compared with non-participants; to local sample	26.7% (36, 37, 43, 44)	
Use of qualitative methods to understand recruitment	20.0% (36, 38, 42)	
**Effectiveness**		
Effectiveness evaluated	73.3% (31, 32, 36–40, 42–45)	In two additional articles, authors report that they did not evaluate effectiveness, but relevant results are reported (34, 35). One article explored effectiveness at the organizational, as well as the individual level (44)
Measure of primary outcome	40.0% (31, 32, 36, 37, 43, 44)	In one additional article, authors highlighted that this evaluation criteria can be challenging and subjective to report on when there are a variety of important outcomes (38)
Measure of primary outcome relative to public health goal	0.0%	In one article where a primary outcome was not identified, outcomes were discussed relative to the Institute of Medicine recommendations on physical activity (39)
Measure of broader outcomes or use of multiple criteria (e.g., measure of quality of life or potential negative outcome)	40.0% (31, 36–40)	
Measure of robustness across subgroups (e.g., moderation analyses)	20.0% (36, 39, 45)	
Measure of short-term attrition (%) and differential rates by patient characteristics or treatment group	6.7% (35)	One article, that had not reported evaluating effectiveness, reported a measure of short-term attrition (%) and differential rates by patient characteristics or treatment group but reported this under reach (35). Two articles (31, 34), one of which had not reported evaluating effectiveness (34), reported a measure of short-term attrition (%) and differential rates by patient characteristics or treatment group, but reported this under adoption (31, 34) Two articles reported a measure of short-term attrition (%) and differential rates by patient characteristics or treatment group, but reported in the Methods section (38, 43)
Use of qualitative methods/data to understand outcomes	20% (32, 38, 42)	
**Adoption-Setting level**		
Setting level adoption evaluated	73.3% (31–35, 38–40, 42, 44, 45)	
Setting exclusions (% or reasons or both)	0.0%	
Percentage of settings approached that participate (valid denominator)	13.3% (31, 33)	The two articles that reported the percentage of settings approached that participated (based on a valid denominator) reported these data under reach (31, 33). An additional article did not report the percentage of settings approached that participated (based on a valid denominator) but provided the data that would allow readers to calculate it (34)
Characteristics of settings participating (both comparison and intervention) compared with either (1) non-participants or (2) some relevant resource data	26.7% (31, 33, 34, 44)	Two of these articles reported this information under reach (31, 33)
Use of qualitative methods to understand setting level adoption	20.0% (33, 34, 42)	For two additional articles it was unclear whether qualitative methods had been used to understand setting level adoption (39, 45)
**Adoption-Staff level**		
Staff level adoption evaluated	60.0% (31, 32, 34–38, 44, 45)	
Staff exclusions (% or reasons or both)	0.0%	
Percent of staff offered that participate	13.3% (36, 37)	
Characteristics of staff participants vs. non-participating staff or typical staff	6.7% (44)	
Use of qualitative methods to understand staff participation/staff level adoption	6.7% (36)	For two additional articles it was unclear whether qualitative methods had been used to understand staff participation/staff level adoption (37, 38)
**Implementation**		
Implementation evaluated	86.7% (31–41, 44, 45)	
Percent of perfect delivery or calls completed (e.g., fidelity)	0.0%	
Adaptations made to intervention during study (not fidelity)	40.0% (34–36, 38, 40, 41)	One of the six articles reported on the adaptations under adoption as well as implementation (35).
Cost of intervention—time	33.3% (31, 37, 38, 41, 44)	One of the five articles reported on length of time for one aspect of the intervention only (41), and one reported the length of time for delivering the intervention separate to RE-AIM results (31)
Cost of intervention—money	26.7% (31, 36, 37, 44)	One of the four articles reported the monetary cost separate to RE-AIM results (31)
Consistency of implementation across staff/time/settings/subgroups (not about differential outcomes, but process)	20.0% (31, 33, 39)	
Use of qualitative methods to understand implementation	66.7% (31–36, 38, 41, 44, 45)	
**Maintenance-Individual level**		
Individual level maintenance evaluated	20.0% (31, 32, 39)	One of the three articles did not distinguish between individual and setting levels and did not report any results relating to maintenance (32)
Measure of primary outcome (with comparison with a public health goal) at ≥6 months follow-up after final treatment contact	0.0%	
Measure of primary outcome ≥6 months follow-up after final treatment contact	6.7% (31)	
Measure of broader outcomes (e.g., measure of quality of life or potential negative outcome) or use of multiple criteria at follow-up	0.0%	
Robustness data—something about subgroup effects over the long-term	0.0%	
Measure of long-term attrition (%) and differential rates by patient characteristics or treatment condition	0.0%	One article reported a measure of long-term attrition (%) under adoption (31)
Use of qualitative methods data to understand long-term effects	0.0%	
**Maintenance-Setting level**		
Setting level maintenance evaluated	60.0% (31–33, 35–38, 40, 45)	
If program is still ongoing at ≥6 months post-treatment follow-up	46.7% (31, 33, 35–38, 45)	An additional article, that did not report on whether the program was maintained 6 months post treatment follow-up, did report the likelihood of maintenance (40)
If and how program was adapted long-term (which elements retained after program completed)	20.0% (31, 33, 35)	One of the three articles, did not systematically collect data on long-term sustainability of the program but reported anecdotal evidence that the program is being maintained and delivery adapted (35)
Some measure/discussion of alignment to organization mission or sustainability of business model	0.0%	
Use of qualitative methods data to understand setting level institutionalization	26.7% (31, 33, 36, 45)	

### Stage Three

#### In-depth Exploration of the Reasoning and Justification for Full and Pragmatic Use of RE-AIM, at the Dimension Level, in a Sub-set of Articles Meeting Inclusion Criteria

The same 15 articles randomly selected for in-depth analysis of RE-AIM application (presented in Stage Two) were further reviewed and data extracted relating to pragmatic application of RE-AIM and challenges and benefits of applying RE-AIM. Table 4 shows justifications for evaluating and not evaluating RE-AIM dimensions, as well as challenges and benefits of applying RE-AIM.

**Table 4:** Details on pragmatic application and challenges and benefits of applying RE-AIM reported across articles included in the sub-analysis.

**References**	**RE-AIM dimensions evaluated**	**Justification for evaluating and/or not evaluating RE-AIM dimension(s)**	**Challenges and benefits of applying RE-AIM**
Aittasalo et al. (31)	R-E-A-I-M	*Justification for evaluating RE-AIM dimension(s)* None reported *Justification for not evaluating RE-AIM dimension(s)* N/A as all RE-AIM dimensions evaluated	None reported
Anderson et al. (32)	R-E-A-I-M	*Justification for evaluating RE-AIM dimension(s)* None reported *Justification for not evaluating RE-AIM dimension(s)* N/A as all RE-AIM dimensions evaluated	None reported
Austin et al. (33)	R-A-I-M	*Justification for evaluating RE-AIM dimension(s)* None reported *Justification for not evaluating RE-AIM dimension(s)* As efficacy for the PA intervention used in this study has been established on a number of previous occasions, this was not the focus of the research	Challenges Furthermore, the modification of the RE-AIM framework (i.e., its application at a setting level vs. both individual and setting levels) posed some challenges. In particular, the differentiation between reach and adoption and the identification of appropriate evaluation measures Benefits However, despite these challenges the framework serves as a good theoretical model for comprehensive public health evaluation, with an emphasis on issues related to external validity
Casey et al. (34)	R-A-I	*Justification for evaluating RE-AIM dimension(s)* Understanding the reach, adoption, and implementation of this program is important to help understand why the program was successful in achieving some of the intended outcomes and why other elements were not achieved *Justification for not evaluating RE-AIM dimension(s)* None reported	None reported
Duffy et al. (35)	R-A-I-M	*Justification for evaluating RE-AIM dimension(s)* None reported *Justification for not evaluating RE-AIM dimension(s)* A prior paper describes the effectiveness of the Tobacco Tactics intervention. This subsequent paper provides data describing the remaining constructs of the RE-AIM framework	Benefits This study has shown how the RE-AIM framework can be used to guide research-based interventions in clinical practice. Utilization of the RE-AIM framework can serve as a guide to plan, conduct, and report on interventions that are implemented on a large scale in real-world settings. Not only can the RE-AIM framework identify individual impact, but it can also identify population impact, as was done in this study. The framework can be used to maximize external validity; report elements of both internal and external validity; review a body of evidence; and compare interventions to make policy decisions As we move toward more population-based interventions, the RE-AIM framework is a valuable guide for implementation
Folta et al. (36)	R-E-A-I-M	*Justification for evaluating RE-AIM dimension(s)* None reported *Justification for not evaluating RE-AIM dimension(s)* N/A as all RE-AIM dimensions evaluated	Challenges A recent systematic review of studies using the RE-AIM framework found that only 44 of 71 articles reported on all five dimensions and that qualitative methods were used very infrequently to provide additional evaluation and understanding. A strength of this study is that the research team received funding just when we were poised to begin major dissemination efforts, and these resources were critical to our ability to evaluate all RE-AIM components using multiple methods. It should be noted, however, that even with substantial planning and resources, our study falls short of meeting all 34 items used to evaluate RE-AIM. For example, we were unable to examine maintenance at the individual level Benefits The RE-AIM framework allowed us to identify strengths as well as areas that might be improved to achieve better public health impact as the program is introduced nationally. It also provides a number of lessons for the translation of similar programs
Folta et al. (37)	R-E-A-I-M	*Justification for evaluating RE-AIM dimension(s)* None reported *Justification for not evaluating RE-AIM dimension(s)* N/A as all RE-AIM dimensions evaluated	Benefits The RE-AIM framework allowed for the identification of strengths and areas needing improvement as national dissemination continues. It also helped identify relevant lessons for similar programs. In conclusion, the RE-AIM framework was valuable in evaluating dissemination and provided several key lessons learned
Jenkinson et al. (38)	R-E-A-I-M	*Justification for evaluating RE-AIM dimension(s)* None reported *Justification for not evaluating RE-AIM dimension(s)* N/A as all RE-AIM dimensions evaluated	Benefits The RE-AIM health promotion evaluation framework was used in this evaluation and has identified a range of different outcomes and limitations that should be considered prior to further implementation and dissemination of the GLAMA program
Lee et al. (39)	R-E-A-I-M	*Justification for evaluating RE-AIM dimension(s)* None reported *Justification for not evaluating RE-AIM dimension(s)* N/A as all RE-AIM dimensions evaluated	None reported
Martinez-Donate et al. (40)	R-E-A-I-M	*Justification for evaluating RE-AIM dimension(s)*] None reported *Justification for not evaluating RE-AIM dimension(s)* N/A as all RE-AIM dimensions evaluated	None reported
Parahoo et al. (41)	I	*Justification for evaluating RE-AIM dimension(s)* None reported *Justification for not evaluating RE-AIM dimension(s)* None reported	None reported
Quinn et al. (42)	R-E-A	*Justification for evaluating RE-AIM dimension(s)* None reported *Justification for not evaluating RE-AIM dimension(s)* None reported	None reported
Ulbricht et al. (43)	R-E	*Justification for evaluating RE-AIM dimension(s)* The dimensions reach and efficacy will be addressed in this paper. Reach refers to the recruitment of a proportion of participants in an intervention among the population of eligible individuals. The reach of socioeconomically disadvantaged populations for interventions has been found to be more likely outside of health care settings and when proactive recruitment within the community setting is used. Given that the home environment has been found to be the primary source of ETS, there may be advantages to the recruitment and delivery of an intervention at the location where children are exposed *Justification for not evaluating RE-AIM dimension(s)* None reported	None reported
Van Acker et al. (44)	R-E-A-I-M	*Justification for evaluating RE-AIM dimension(s)* None reported *Justification for not evaluating RE-AIM dimension(s)* N/A as all RE-AIM dimensions evaluated	None reported
Wallace et al. (45)	R-E-A-I-M	*Justification for evaluating RE-AIM dimension(s)* None reported *Justification for not evaluating RE-AIM dimension(s)* N/A as all RE-AIM dimensions evaluated	None reported

*Justification for/for not evaluating RE-AIMS dimension(s)*.

*Green, Justification reported; Blue, Justification not presented; Orange, Not applicable at all RE-AIM dimensions evaluated (all text directly copied from full text article)*.

*Challenges and benefits of applying RE-AIM*.

*Green, Benefits; Red, challenges; Orange, None reported (all text directly copied from full text article)*.

##### Justification for Evaluating and/or Not Evaluating RE-AIM Dimension(s)

Twelve/fifteen articles did not justify the rationale for choosing to evaluate particular RE-AIM dimension(s) (31, 32, 35–42, 44, 45).

Nine/fifteen articles evaluated all RE-AIM dimensions (31, 32, 36–40, 44, 45), therefore justifying the rationale for choosing *not* to evaluate particular RE-AIM dimensions was not applicable. Of the six articles that chose *not* to evaluate one or more RE-AIM dimension, four articles did not justify the rationale for choosing *not* to evaluate particular RE-AIM dimensions (34, 41–43).

##### Challenges and Benefits of Applying RE-AIM

Ten/Fifteen articles did not report any challenges or benefits of applying RE-AIM (31, 32, 34, 39–45). Two/Fifteen articles reported challenges of applying RE-AIM (33, 36). Challenges reported included differentiating between reach and adoption dimensions and the identification of appropriate evaluation measures (33), as well as the challenge of evaluating all 34 evaluation criteria, despite substantial planning and resources (36). Five/Fifteen articles reflected and reported on the benefits of applying RE-AIM (33, 35–38). Reported benefits of applying RE-AIM included that the framework serves as a good theoretical model for comprehensive public health evaluation, with an emphasis on issues related to external validity (33) and that RE-AIM can identify individual, as well as population, impact (35).

## Discussion

This is the first systematic review of the use of RE-AIM across countries, categorized according to income, and the first systematic assessment of the pragmatic application and rationale for pragmatic use of the framework. We found that RE-AIM has predominantly been applied in high-income countries, with very few applications in middle-income, lower-middle and lower-income economy countries. We found that RE-AIM is frequently applied pragmatically (i.e., partial application of the framework). However, when applied pragmatically, authors do not always provide justification for pragmatic application.

Reach was the most frequently reported dimension, followed by implementation, adoption, effectiveness, and maintenance. RE-AIM is predominantly applied as an evaluation framework, with very few studies using the framework as a planning or planning and evaluation framework.

Comparing the results of this review to that conducted by Gaglio et al. (14) we found several similarities. We found that RE-AIM continues to be applied across a diverse range of topic areas. We found a similar proportion of studies evaluating one, two, three, four, and five RE-AIM dimensions across the two reviews. In both reviews, reach was the most frequently evaluated RE-AIM dimension and maintenance was the least frequently evaluated dimension. Of note, we found a relatively large increase in the number of studies evaluating adoption compared to Gaglio et al. (14). Comparing the mean yearly publication rate, between 1999–2010, and 2011–2017 (i.e., the two review periods), we found an increase from 6.5 to 22.4 studies.

The in-depth analysis of RE-AIM application at a dimension and evaluation criteria level, in the sub-set of articles, revealed significant variation in which evaluation criteria were reported. Several evaluation criteria were not reported in any of the articles included in the sub-analyses. These included, but were not limited to, (i) the percentage of individuals that were excluded or the characteristics of those that were excluded, for reach, (ii) measurement of a primary outcome relative to a public health goal, for effectiveness, (iii) the percentage of staff exclusions or reasons why staff were excluded, for adoption, (iv) the percentage of perfect delivery or calls completed, for fidelity, and (v) robustness data relating to subgroup effects over the long-term, for maintenance. Interestingly, Gaglio et al. (14) did not identify any evaluation criteria that were not reported on in any of the included articles. The evaluation criteria least frequently reported on in their review were those relating to the use of qualitative methods to understand setting level adoption, as well as the percentage of staff exclusions or reasons why staff were excluded, for adoption, and if and how a program was adapted long term, for maintenance.

We found multiple instances in which evaluation criteria were reported under the incorrect RE-AIM dimension [e.g., reporting the percentage of settings approached that participated (based on a valid denominator) under reach instead of adoption]. Some evaluation criteria had also been operationalised differently by authors [e.g., at a different level (e.g., reporting the percentage of individuals, who participate, based on a valid denominator, at the school rather than individual level) or using different methods (e.g., reporting on likelihood of maintenance or anecdotal evidence that the program is being maintained and delivery adapted rather than reporting undisputable data regarding whether the program had been maintained)]. Some of these findings are similar to the “problems” identified by Gaglio et al. (14) for example, confusing the definitions of reach and adoption. They also reflect issues acknowledged in the recent guidance from the RE-AIM developers regarding pragmatic use of the framework (13) and a recent publication that sought to improve understanding and application of RE-AIM by identifying common misconceptions (categorized as “conceptual issues,” “methodological issues,” and “use of the model issues”) and providing guidance to overcome them (46).

The use of qualitative methods/data to understand RE-AIM dimensions, in the present review, was employed for all dimensions except to understand long-term effects (maintenance at the individual level). This has been actively encouraged in recent guidance and represents a positive development in the application of RE-AIM (13, 46). However, it is notable that the reporting of some evaluation criteria, particularly those relating to health equity, was limited and would benefit from improvement. For example, evaluation criteria relating to representativeness (e.g., characteristics of participants compared with non-participants; to local sample) were only reported in between 0 and 26.7% of studies across the RE-AIM dimensions. Furthermore, reporting of other evaluation criteria, that may be of significant interest to implementation stakeholders, was also limited. For example, only 40% of studies reported whether adaptations were made to the intervention during study and just 33% reported the time required to implement.

Despite variation in application of the framework and multiple examples of pragmatic application, few articles included in our sub-analyses explicitly reported the reasons behind pragmatic use. As with adaptations to interventions, adaptations to applications of TMFs are not inherently problematic (and in some cases are encouraged) but they need to be explicitly reported in order for results to be interpreted appropriately and opportunities for refinements to both the intervention(s) and the TMFs to be maximized. A lack of reporting makes it difficult to distinguish between pragmatic application (with appropriate justification) and key issues being overlooked (26) and superficial and mis-application (7, 46). More generally, the authors of the studies included in this review did not typically reflect on the challenges or benefits of applying RE-AIM. This lack of reporting was particularly notable in relation to authors reflecting on the challenges of applying the framework. Reporting of challenges and benefits associated with use should be encouraged as this can help to refine TFMs, as well as prompt TFM developers to provide guidance on how best to overcome challenges in application [e.g., see (13, 46, 47)].

### Implications and Recommendations

In line with previous recommendations (14), we recommend that if RE-AIM is applied pragmatically (i.e., not in its full form), investigators provide justification for evaluating the selected RE-AIM dimensions and also justification for not evaluating RE-AIM dimensions. It is important to note that while two of the most recent and detailed articles advocating the pragmatic use of RE-AIM were published in 2018 and 2019 (13, 26), in 2013, Gaglio et al. (14) proposed that not all RE-AIM dimensions must be used in all studies, but recommended that investigators be clear on what elements of the framework are used and why these were selected or not. Thus, it is reasonable to expect investigators to describe and justify pragmatic application of RE-AIM prior to the 2018 and 2019 publications (13, 26), and we found this to be the case in a number of articles included in this review. However, it is not reasonable to expect investigators applying RE-AIM pragmatically to have addressed all the recommendations included in the more recent articles. Our position is that there are often scientific and/or pragmatic reasons why investigators do not apply RE-AIM in its full form, but unless these reasons are clearly articulated, it is not possible to rule out that key implementation issues have been overlooked or feed findings back into refinements of the framework. These recommendations are not unique to RE-AIM and are relevant to other TMFs that are being applied pragmatically. Future work should explore pragmatic use of other TMFs and the extent to which they are reported appropriately in the literature. Given that RE-AIM has been predominately applied in high-income countries, we recommend that researchers and practitioners applying the framework in lower income countries (including upper-middle-income, lower-middle, or low-income countries) reflect upon and report on the utility of the framework in these settings. Furthermore, given that RE-AIM has been predominately used as an evaluation framework, we recommend that researchers and practitioners applying RE-AIM as a planning framework, reflect upon and report on the utility of the framework for this purpose.

We experienced significant challenges as the associated evaluation criteria relating to each RE-AIM dimension were not explicitly operationalised in the Gaglio et al. review. Therefore, the comparisons we draw between stage two results of this review and the results reported by Gaglio et al. should be interpreted with a degree of caution. We recommend that, in the future, the way in which RE-AIM evaluation criteria are operationalised is clearly articulated to allow for comparisons to be made between studies and across reviews. Recent guidance has acknowledged the challenges in operationalisation and offered clarifications and resources (46).

### Strengths and Limitations

Strengths of this review include the three-stage process which enabled exploration of the articles from multiple perspectives in line with four distinct, but related research objectives. Furthermore, significant accuracy and reliability checks were incorporated into each step of data screening and extraction to ensure quality. This is the first systematic review of the use of RE-AIM across countries, categorized according to income, and the first systematic assessment of the pragmatic application and rationale for use of the framework.

There are several limitations to our review that must be noted. Due to capacity within our team, we were only able to conduct an in-depth exploration of the pragmatic use of RE-AIM and reasoning and justification for pragmatic use, in a sub-set of articles meeting inclusion criteria. In total, the sub-set of articles reviewed consisted of 15 articles. Ideally, this would have been done on all included articles and not doing so limits the confidence in comparisons drawn with the findings of the Gaglio et al. review, and the conclusions drawn here. However, given the capacity of our research team, we were only able to focus on a smaller sample for in-depth exploration.

A further limitation is that the search period was up to end of 2017. More recent advances in how the RE-AIM framework is being used and reported on would not have been captured. Specifically, we recommend that future research explores whether investigators that have applied RE-AIM since 2019, i.e., after the publication of the two most recent and detailed articles advocating pragmatic use of RE-AIM (13, 26), have adhered to the recommendations relating to pragmatic use of RE-AIM. It is possible that the pragmatic use of RE-AIM has changed and advanced since these two more recent publications (13, 26). However, comparing our findings with those reported by Gaglio et al. (14) there are many striking similarities which indicates that these concerns may not be valid. Future work should explore more recent applications of the framework, which is particularly relevant in light of recent attempts to provide additional support and guidance and overcome misconceptions (46).

Furthermore, we did not extract outcome data relating to each RE-AIM dimension and associated evaluation criteria from the included articles. Therefore, we are unable to draw any conclusions regarding the impact of the interventions under study and more wide-ranging issues that could have been uncovered if we had extracted outcome data, such as those relating to health equity. However, our review was focused on how RE-AIM is being applied and reported in the literature.

## Conclusions

An increasing number of studies are applying the RE-AIM framework to plan and evaluate the implementation of a diverse range of healthcare and non-healthcare interventions. However, there is evidence that RE-AIM is frequently not applied in its full form (i.e., it is applied pragmatically), yet the reasons provided for this are minimal. This lack of reporting limits opportunities to monitor the quality of application as well as feed findings back into future refinement of the framework.

## Data Availability Statement

The original contributions presented in the study are included in the article/Supplementary Material, further inquiries can be directed to the corresponding author/s.

## Author Contributions

DD'L and LH conceptualized, designed the study, screened citations for inclusion, drafted the manuscript, and extracted data at all stages. TS contributed to data extraction at stage one and reviewed and edited the draft manuscript. All authors read and approved the final version of the manuscript.

## Funding

LH, King's College London, is supported by the National Institute for Health Research (NIHR) Applied Research Collaboration South London (NIHR ARC South London) at King's College Hospital NHS Foundation Trust. LH is a member of King's Improvement Science, which offers co-funding to the NIHR ARC South London and comprises a specialist team of improvement scientists and senior researchers based at King's College London. Its work is funded by King's Health Partners (Guy's and St Thomas' NHS Foundation Trust, King's College Hospital NHS Foundation Trust, King's College London and South London and Maudsley NHS Foundation Trust), Guy's and St Thomas' Charity, and the Maudsley Charity. The views expressed are those of the author[s] and not necessarily those of the NIHR or the Department of Health and Social Care. The funders were not involved in the design, conduct or reporting of the study. TS research was supported by the Wellcome Trust (grant number 219425/Z/19/Z) and Diabetes UK (grant number 19/0006055).

## Author Disclaimer

The views expressed in this publication are those of the authors and not necessarily those of King's Health Partners, the NIHR, the NHS, or the Department of Health and Social Care.

## Conflict of Interest

TS received funding from Cancer Alliances, NHS England, and Health Education England for training cancer multidisciplinary teams in assessment and quality improvement methods in the United Kingdom. TS also received fees from Roche Diagnostics for research services in relation to implementation and evaluation of innovations for cancer multidisciplinary teams in the United States of America. The remaining authors declare that the research was conducted in the absence of any commercial or financial relationships that could be construed as a potential conflict of interest.

## Publisher's Note

All claims expressed in this article are solely those of the authors and do not necessarily represent those of their affiliated organizations, or those of the publisher, the editors and the reviewers. Any product that may be evaluated in this article, or claim that may be made by its manufacturer, is not guaranteed or endorsed by the publisher.
