# Supplementary material for: Evaluating the Application of the RE-AIM Planning and Evaluation Framework: An Updated Systematic Review and Exploration of Pragmatic Application
Source: Front Public Health. 2022 Jan 26;9:755738. doi: 10.3389/fpubh.2021.755738 (PMC8826088; doi:10.3389/fpubh.2021.755738)
Supplement: Supplementary file 1 [file Table_1.docx]

**Supplementary File 1: Detailed overview of protocol deviations**

| **Information reported in protocol** | **Deviation(s)** | **Reason for deviation(s)** |
| --- | --- | --- |
| **Review questions**   1. To update the systematic review conducted by Gaglio et al, 2013^1^ to provide a comparison of the use of the RE-AIM framework over time, specifically comparing use over two time periods; 1999-2010 and 2011-2016 2. To explore whether the recommendations for RE-AIM applications identified by Gaglio et al, 2013^1^ have been adhered to 3. To explore whether RE-AIM is being used in conjunction with other implementation theories, frameworks and models | Review question one was completed at the dimension level only. Item level extraction was completed on a sub-set of articles meeting inclusion criteria. The time period was extended to include all articles published in 2017.  Review question two was considered informally as item level extraction was completed on a sub-set of articles.  Review question three was replaced with an in-depth exploration of the reasoning and justification for full and pragmatic use of RE-AIM, at a dimension level, in a sub-set of articles meeting inclusion criteria.  The revised review aims were as follows:   1. To document the evolution of RE-AIM application by providing an updated synthesis of RE-AIM use from 2011-2017. 2. To compare the results to a systematic review of RE-AIM use over time from 1999-2010, published by Gaglio et al, 2013^1^ 3. To provide an in-depth exploration of the pragmatic use of RE-AIM at a (1) dimension level (e.g., reach) and (2) evaluation criteria level (e.g., exclusion criteria (% excluded or characteristics), in a sub-set of articles meeting inclusion criteria. 4. To provide an in-depth exploration of the reasoning and justification for full and pragmatic use of RE-AIM, at a dimension level, in a sub-set of articles meeting inclusion criteria and document the challenges and benefits of applying RE-AIM reported by authors. | The focus of the review was adapted to reflect more recent developments in understanding and evaluating the application of RE-AIM, and also the changing capacity of the review team (40% reduction). |
| **Searches**  The following databases will be searched: MEDLINE (R) and PsycINFO, searched via the Ovid interface. Scopus and Web of Science Core Collection will be used for citation tracking.  RE-AIM will be used as a keyword.  The following restrictions will be applied: English language; publication period: 01/01/2011-31/12/2016. To ensure all relevant studies are identified, the reference lists of included studies and relevant reviews identified in the search will be scanned. | We did not use Scopus and Web of Science Core Collection for citation tracking and reference lists were not scanned.  The time period was extended to include articles published in 2017. | This was not completed due to the large number of citations retrieved and the changing capacity of the review team (40% reduction). |
| **Types of study to be included**  Qualitative, quantitative, mixed-method studies will be eligible for inclusion. Exclusions: commentaries, theoretical papers, published abstracts (including conference proceedings), dissertations, book chapters, editorials, research protocols, or did not report empirical or evaluative data on the use of RE-AIM for planning or evaluation of a study, program, or policy.  **Condition or domain being studied**  Healthcare (including all diseases and conditions) and non-healthcare domains that plan and/or evaluate a study, program or policy using the RE-AIM framework will be eligible for inclusion.  **Participants/population**  All participant and population groups will be eligible for inclusion.  **Intervention(s), exposure(s)**  Any study, program or policy that has been planned or evaluated using the Reach, Effectiveness, Adoption, Implementation, and Maintenance (RE-AIM) framework.  **Comparator(s)/control**  Not applicable  **Context**  All study settings will be included in the review.  **Main outcome(s)**  This review will explore the application of the RE-AIM framework to a given intervention or program across all of its domains, namely: Reach, Effectiveness, Adoption, Implementation, and Maintenance. Therefore a primary outcome is not applicable.  **Measures of effect**  Not applicable  **Additional outcome(s)**  None  **Measures of effect**  Not applicable | No deviations to report. | N/A |
| **Data to be extracted:**  • Author  • Date of publication  • Journal name  • Study design  • Phase of implementation RE-AIM applied (pre-, during-, post-implementation)  • Intervention/program  • Intervention level (e.g. population)  • Research setting (e.g. primary care)  • Content area (e.g. health promotion)  • Reach (4 criteria): Exclusion criteria (% excluded or characteristics); Percentage of individuals who participate, based on valid denominator; Characteristics of participants compared with nonparticipants; to local sample; Use of qualitative methods to understand recruitment  • Effectiveness (6 criteria): Measure of primary outcome; Measure of primary outcome relative to public health goal; Measure of broader outcomes or use of multiple criteria (e.g., measure of quality of life or potential negative outcome); Measure of robustness across subgroups (e.g., moderation analyses); Measure of short-term attrition (%) and differential rates by patient characteristics or treatment group; Use of qualitative methods/data to understand outcomes  • Adoption-setting level (4 criteria): Setting exclusions (% or reasons or both); Percentage of settings approached that participate (valid denominator); Characteristics of settings participating (both comparison and intervention) compared with either (1) non-participants or (2) some relevant resource data; Use of qualitative methods to understand setting level adoption  • Adoption-staff level (4 criteria): Staff exclusions (% or reasons or both); Percent of staff offered that participate; Characteristics of staff participants vs non-participating staff or typical staff; Use of qualitative methods to understand staff participation/staff level adoption  • Implementation (6 criteria): Percent of perfect delivery or calls completed (e.g., fidelity); Adaptations made to intervention during study (not fidelity); Cost of intervention-time; Cost of intervention-money; Consistency of implementation across staff/time/settings/subgroups (not about differential outcomes, but process); Use of qualitative methods to understand implementation  • Maintenance-individual level (6 criteria): Measure of primary outcome (with comparison with a public health goal) at ‡ 6 month follow-up after final treatment contact; Measure of primary outcome ‡ 6 month follow-up after final treatment contact; Measure of broader outcomes (e.g., measure of quality of life or potential negative outcome) or use of multiple criteria at follow-up; Robustness data—something about subgroup effects over the long-term; Measure of long-term attrition (%) and differential rates by patient characteristics or treatment condition; Use of qualitative methods data to understand long-term effects  • Maintenance-setting level (4 criteria): If program is still ongoing at ‡ 6 mo post-treatment follow-up; If and how program was adapted long-term (which elements retained after program completed); Some measure/discussion of alignment to organization mission or sustainability of business model; Use of qualitative methods data to understand setting level institutionalization  • Number of RE-AIM dimensions assessed  • Number of criteria assessed (out of 34)  • Justification for using RE-AIM and dimensions assessed  • Other implementation theories, frameworks, models used in conjunction with RE- AIM  • Authors thoughts on applying RE-AIM (+ & -) | The following data were extracted for all articles meeting inclusion criteria:   - Author - Date of publication - Journal name - Study design - Topic area - Country in which the study was conducted, - Nature of RE-AIM use - Whether the authors reported evaluating (1) reach, (2) effectiveness, (3) adoption, (4) implementation, (5) maintenance, number of RE-AIM dimensions evaluated and RE-AIM dimensions combination.   Please see Supplementary File 2 for the items that were extracted on for the in-depth exploration of the pragmatic use of RE-AIM at a dimension and item level, in a sub-set of articles meeting inclusion criteria. | These changes were in line with our revised review questions which were adapted to reflect more recent developments in understanding and evaluating the application of RE-AIM, and also the changing capacity of the review team (40% reduction). |
| **Risk of bias (quality) assessment**  This review will explore the application of the RE-AIM framework to a given intervention or program. The findings of the review will summarise the extent to which the framework has been applied, rather than summarise the findings of the included studies per se. Therefore it is not necessary to conduct a bias assessment of the included studies | No deviations to report. | N/A |
| **Strategy for data synthesis**  A narrative synthesis is planned. | No deviations to report. | N/A |
| **Analysis of subgroups or subsets**  None planned | An in-depth exploration of the pragmatic use of RE-AIM at a dimension and item level, in a sub-set of articles meeting inclusion criteria was conducted. This was followed by an in-depth exploration of the reasoning and justification for full and pragmatic use of RE-AIM, at a dimension level, in the same sub-set of articles meeting inclusion criteria. | The focus of the review was adapted to reflect more recent developments in understanding and evaluating the application of RE-AIM |
| **Review team members and their organisational affiliations**  Dr Louise Hull. King's College London  Dr Rachel Davis. King's College London  Ms Charlotte Rowland. King's College London  Dr Danielle D'Lima. University College London  Dr Zarnie Khadjesari. King's College London | Review team members reduced to three people only.  Dr Danielle D'Lima. University College London  Dr Tayana Soukup. King's College London (new team member)  Dr Louise Hull. King's College London | The reduced capacity of the review team was due to job role changes. |

**References**

1. Gaglio B, Shoup JA, Glasgow RE. The RE-AIM framework: a systematic review of use over time. *Am J Public Health.* 2013;103(6):e38-46.
