# Supplementary material for: Evaluating the Application of the RE-AIM Planning and Evaluation Framework: An Updated Systematic Review and Exploration of Pragmatic Application
Source: Front Public Health. 2022 Jan 26;9:755738. doi: 10.3389/fpubh.2021.755738 (PMC8826088; doi:10.3389/fpubh.2021.755738)
Supplement: Supplementary file 2 [file Table_2.docx]

**Supplementary File 2. Data extraction table: Criteria and operational definitions**

| **Section 1: Criteria relating to the key characteristics of the article** | | |
| --- | --- | --- |
| First author | | First author of article |
| Year of publication | | Date article published |
| Journal | | Name of journal that article is published in |
| Country | | Country/countries where study took place |
| Income classification of country | | Income classification of country based on the 2018 World Bank classification criteria. Available from: <https://datahelpdesk.worldbank.org/knowledgebase/articles/906519-world-bank-country-and-lending-groups> . Last accessed 12th April 2021 |
| Study design | | Study design (quantitative, qualitative or mixed-method) |
| **Criteria pertaining to the high-level application of RE-AIM** | | |
| Topic area | | Brief description of topic area e.g., physical activity |
| Nature of RE-AIM application | | Nature of RE-AIM application (planning, evaluation or planning and evaluation) |
| Reach evaluated | | Whether authors report evaluating the reach dimension of RE-AIM, regardless of whether authors report results relating to reach |
| Effectiveness evaluated | | Whether authors report evaluating the effectiveness dimension of RE-AIM, regardless of whether authors report results relating to effectiveness |
| Adoption evaluated | | Whether authors report evaluating the adoption dimension of RE-AIM, regardless of whether authors report results relating to adoption |
| Implementation evaluated | | Whether authors report evaluating the implementation dimension of RE-AIM, regardless of whether authors report results relating to implementation |
| Maintenance evaluated | | Whether authors report evaluating the maintenance dimension of RE-AIM, regardless of whether authors report results relating to maintenance |
| RE-AIM combinations | | The combination of RE-AIM dimensions that authors report evaluating, regardless of whether authors report results relating to each dimension- e.g., R-I-M would indicate that authors report evaluating reach, implementation, and maintenance |
| Number of RE-AIM dimensions evaluated | | The number of RE-AIM dimensions that authors report evaluating |
| **Section 2: Criteria relating to reporting at the RE-AIM dimension criteria level** | | |
| REACH | | |
| Exclusion Criteria (% excluded or characteristics) | Whether authors report either the percentage of individuals excluded or the characteristics of those excluded (yes/no/unclear) | |
| Percentage of individuals, who participate, based on valid denominator | Whether authors report the percentage of individuals, who participate, based on a valid denominator (yes/no/unclear) | |
| Characteristics of participants compared with nonparticipants; to local sample | Whether authors report characteristics of participants compared to either nonparticipants or a local sample (yes/no/unclear) | |
| Use of qualitative methods to understand recruitment | Whether authors use qualitative methods to understand recruitment (yes/no/unclear) | |
| EFFECTIVENESS | | |
| Measure of primary outcome | Whether authors explicitly identify a primary or set of primary outcomes (yes/no/unclear) | |
| Measure of primary outcome relative to public health goal | Whether authors measure the primary outcome(s) relative to a public health goal (yes/no/unclear) | |
| Measure of broader outcomes or use of multiple criteria (e.g., measure of quality of life or potential negative outcome) | Whether authors measure broader outcomes or use of multiple criteria (e.g., measure of quality of life or potential negative outcome) (yes/no/unclear) | |
| Measure of robustness across subgroups (e.g., moderation analyses) | Whether authors measure robustness across subgroups (e.g., moderation analyses) (yes/no/unclear) | |
| Measure of short-term attrition (%) and differential rates by patient characteristics or treatment group | Whether authors measure short-term attrition (%) and differential rates by either patient characteristics or treatment group (yes/no/unclear) | |
| Use of qualitative methods/data to understand outcomes | Whether authors use qualitative methods/data to understand outcomes (yes/no/unclear) | |
| ADOPTION | | |
| *Setting Level* | | |
| Setting exclusions (% or reasons or both) | Whether authors report either the percentage of settings excluded or the reasons for exclusion or both (yes/no/unclear) | |
| Percentage of settings approached that participate (valid denominator) | Whether authors report the percentage of settings approached, that participate, based on a valid denominator (yes/no/unclear) | |
| Characteristics of settings participating (both comparison and intervention) compared with either (1) nonparticipants or (2) some relevant resource data | Whether authors report characteristics of settings participating compared to either nonparticipants or some relevant resource data (yes/no/unclear) | |
| Use of qualitative methods to understand setting level adoption | Whether authors use qualitative methods to understand setting level adoption (yes/no/unclear) | |
| *Staff Level* | | |
| Staff exclusions (% or reasons or both) | Whether authors report either the percentage of staff excluded or the reasons for exclusion or both (yes/no/unclear) | |
| Percent of staff offered that participate | Whether authors report the percentage of staff offered that participate (yes/no/unclear) | |
| Characteristics of staff participants vs nonparticipating staff or typical staff | Whether authors report characteristics of staff participating compared to either nonparticipating staff or typical staff (yes/no/unclear) | |
| Use of qualitative methods to understand staff participation/staff level adoption | Whether authors use qualitative methods to understand staff participation/staff level adoption (yes/no/unclear) | |
| IMPLEMENTATION | | |
| Percent of perfect delivery or calls completed (e.g., fidelity) | Whether authors report percent of perfect delivery or calls completed (e.g., fidelity) (yes/no/unclear) | |
| Adaptations made to intervention during study (not fidelity) | Whether authors report if adaptations were made to the intervention during study (not fidelity) (yes/no/unclear) | |
| Cost of intervention—time | Whether authors report cost of the intervention in time (yes/no/unclear) | |
| Cost of intervention—money | Whether authors report cost of the intervention in money (yes/no/unclear) | |
| Consistency of implementation across staff/time/settings/subgroups (not about differential outcomes, but process) | Whether authors report consistency of implementation across staff/time/settings/subgroups (not about differential outcomes, but process) (yes/no/unclear) | |
| Use of qualitative methods to understand implementation | Whether authors use qualitative methods to understand implementation (yes/no/unclear) | |
| MAINTENANCE | | |
| *Individual Level* | | |
| Measure of primary outcome (with comparison with a public health goal) at ≥ 6 mo follow-up after final treatment contact | Whether authors measure the primary outcome(s) (with comparison with a public health goal) at either 6-month follow-up after final treatment contact or a longer period (yes/no/unclear) | |
| Measure of primary outcome ≥ 6 mo follow-up after final treatment contact | Whether authors measure the primary outcome(s) at either 6-month follow-up after final treatment contact or a longer period (yes/no/unclear) | |
| Measure of broader outcomes (e.g., measure of quality of life or potential negative outcome) or use of multiple criteria at follow-up | Whether authors measure broader outcomes (e.g., measure of quality of life or potential negative outcome) or use of multiple criteria at follow-up (yes/no/unclear) | |
| Robustness data—something about subgroup effects over the long-term | Whether authors report robustness data on subgroup effects over the long-term (yes/no/unclear) | |
| Measure of long-term attrition (%) and differential rates by patient characteristics or treatment condition | Whether authors measure long-term attrition (%) and differential rates by patient characteristics or treatment condition (yes/no/unclear) | |
| Use of qualitative methods/data to understand long-term effects | Whether authors use qualitative methods/data to understand long-term effects (yes/no/unclear) | |
| *Setting Level* | | |
| If program is still ongoing at ≥ 6 mo posttreatment follow-up | Whether authors report if the program is still ongoing at 6-month posttreatment follow-up or later (yes/no/unclear) | |
| If and how program was adapted long-term (which elements retained after program completed) | Whether authors report if and how the program was adapted long-term (which elements were retained after program was completed) (yes/no/unclear) | |
| Some measure/discussion of alignment to organisation mission or sustainability of business model | Whether authors report some measure/discussion of alignment to organisation mission or sustainability of business model (yes/no/unclear) | |
| Use of qualitative methods/data to understand setting level institutionalisation | Whether authors use qualitative methods/data to understand setting level institutionalisation (yes/no/unclear) | |
| **Section 3: Criteria relating to pragmatic application of RE-AIM** | | |
| Justification for evaluating and/or not evaluating RE-AIM dimension(s) | Extract any data from the article which provides justification for evaluating and/or not evaluating RE-AIM dimension(s) | |
| Challenges and benefits of applying RE-AIM | Extract any data from the article which reports challenges and/or benefits of applying RE-AIM | |
