# Supplementary material for: Evaluating the Application of the RE-AIM Planning and Evaluation Framework: An Updated Systematic Review and Exploration of Pragmatic Application
Source: Front Public Health. 2022 Jan 26;9:755738. doi: 10.3389/fpubh.2021.755738 (PMC8826088; doi:10.3389/fpubh.2021.755738)
Supplement: Supplementary file 4 [file Table_4.docx]

**Supplementary file 4: Country and income classification of included studies**

| **First author, date of publication** | **Country** | **Income classification*** |
| --- | --- | --- |
| Adams, 2017[1] | UK | High-income |
| Aerts, 2013[2] | Belgium | High-income |
| Aittasalo, 2012[3] | Finland | High-income |
| Allicock, 2013[4] | USA | High-income |
| Altpeter, 2015[5] | USA | High-income |
| Anderson, 2015[6] | USA | High-income |
| Anderson, 2017[7] | USA | High-income |
| Anesetti-Rothermel, 2012[8] | USA | High-income |
| Arbour-Nicitopoulos, 2014[9] | Canada | High-income |
| Arrossi, 2017[10] | Argentina | Upper-middle-income |
| Austin, 2011[11] | Australia | High-income |
| Baba, 2017[12] | Brazil | Upper-middle income |
| Banfield, 2015[13] | Australia | High-income |
| Belkora, 2015[14] | USA | High-income |
| Bhardwaja, 2011[15] | USA | High-income |
| Bhojani, 2015[16] | India | Lower-middle income |
| Boersma, 2017[17] | Netherlands | High-income |
| Brace, 2015[18] | USA | High-income |
| Brinkley, 2017[19] | UK | High-income |
| Brown, 2016[20] | South Africa | Upper-middle-income |
| Broyles, 2013[21] | USA | High-income |
| Brunisholz, 2017[22] | USA | High-income |
| Buis, 2013[23] | USA | High-income |
| Bukhari, 2011[24] | USA | High-income |
| Burke, 2015[25] | Canada | High-income |
| Caperchione, 2016[26] | Australia | High-income |
| Carlfjord, 2012[27] | Sweden | High-income |
| Carlfjord, 2013[28] | Sweden | High-income |
| Casey, 2014[29] | Australia | High-income |
| Chao, 2015[30] | USA | High-income |
| Chen, 2015[31] | Canada | High-income |
| Christian, 2016[32] | UK | High-income |
| Clemson, 2014[33] | Australia | High-income |
| Conlon, 2015[34] | USA | High-income |
| Conte, 2016[35] | USA | High-income |
| Cook, 2013[36] | USA | High-income |
| Damschroder, 2017[37] | USA | High-income |
| Dubuy, 2013[38] | Belgium | High-income |
| Duffy, 2015[39] | USA | High-income |
| Duffy, 2016[40] | USA | High-income |
| Dunton, 2014[41] | USA | High-income |
| Eisen, 2013[42] | USA | High-income |
| Elliot, 2014[43] | Australia | High-income |
| Estabrook, 2012[44] | USA | High-income |
| Evenson, 2013[45] | USA | High-income |
| Finocchario-Kessler, 2015[46] | Kenya | Lower-middle income |
| Folta^a^, 2015[47] | USA | High-income |
| Folta^b^, 2015[48] | USA | High-income |
| Fortney, 2012[49] | USA | High-income |
| Fortney, 2013[50] | USA | High-income |
| Freyer-Adam, 2016[51] | Germany | High-income |
| Gainforth, 2015[52] | Canada | High-income |
| Glasgow, 2011[53] | USA | High-income |
| Glasgow, 2013[54] | USA | High-income |
| Goode, 2013[55] | Australia | High-income |
| Gordon, 2012[56] | USA | High-income |
| Grow, 2014[57] | USA | High-income |
| Hagedorn, 2014[58] | USA | High-income |
| Hanson, 2014[59] | USA | High-income |
| Harden, 2014[60] | USA | High-income |
| Harden, 2017[61] | USA | High-income |
| Headley, 2013[62] | USA | High-income |
| Huang, 2015[63] | Taiwan | High-income |
| Huang, 2017[64] | Uganda | Low-income |
| Hussain, 2016[65] | USA | High-income |
| Huye, 2014[66] | USA | High-income |
| Hynes, 2017[67] | USA | High-income |
| Jaipakdee, 2015[68] | Thailand | Upper-middle-income |
| Jang, 2015[69] | USA | High-income |
| Janssen, 2013[70] | Netherlands | High-income |
| Jenkinson, 2012[71] | Australia | High-income |
| Jenny, 2015[72] | Switzerland | High-income |
| Jeong, 2015[73] | Korea | High-income |
| Jones, 2016[74] | Australia | High-income |
| Kahwati, 2011[75] | USA | High-income |
| Kastirke, 2013[76] | Germany | High-income |
| Kim, 2012[77] | USA | High-income |
| Kirchner, 2014[78] | USA | High-income |
| Koorts, 2015[79] | UK | High-income |
| Kozica, 2016[80] | Australia | High-income |
| Krist, 2014[81] | USA | High-income |
| Lane, 2015[82] | UK | High-income |
| Larsen, 2017[83] | USA | High-income |
| Larsen, 2015[84] | USA | High-income |
| Lee, 2019[85] | USA | High-income |
| Leenaars, 2017[86] | Netherlands | High-income |
| Leveille, 2012[87] | USA | High-income |
| Lewis, 2017[88] | USA | High-income |
| Li, 2013[89] | USA | High-income |
| Liddy, 2016[90] | Canada | High-income |
| Lindblom, 2014[91] | Sweden | High-income |
| Liu, 2015[92] | USA | High-income |
| Lum, 2016[93] | USA | High-income |
| Mahabee-Gittens, 2014[94] | USA | High-income |
| Martinez-Donate, 2015[95] | USA | High-income |
| Maxwell, 2016[96] | USA | High-income |
| Meyer, 2012[97] | Germany | High-income |
| Mielenz, 2014[98] | USA | High-income |
| More, 2017[99] | India | Lower-middle income |
| Nigg, 2012[100] | USA | High-income |
| Nothwehr, 2014[101] | USA | High-income |
| Odgers-Jewell, 2017[102] | Australia | High-income |
| Oldroyd, 2017[103] | Australia | High-income |
| Olstad, 2016[104] | Australia | High-income |
| Paez, 2015[105] | Brazil and Columbia | Upper-middle income and upper-middle income |
| Palacio, 2016[106] | USA | High-income |
| Paone, 2014[107] | USA | High-income |
| Parahoo, 2017[108] | Ireland | High-income |
| Patel, 2013[109] | Australia & USA | High-income & high income |
| Payne, 2011[110] | Australia | High-income |
| Peels, 2012[111] | Netherlands | High-income |
| Peralta, 2017[112] | USA | High-income |
| Perry, 2017[113] | Australia | High-income |
| Poulos, 2012[114] | Australia | High-income |
| Poulos, 2015[115] | Australia | High-income |
| Quinn, 2015[116] | USA | High-income |
| Rasmussen, 2014[117] | Denmark | High-income |
| Resnick, 2013[118] | USA | High-income |
| Resnick, 2014[119] | USA | High-income |
| Samia, 2014[120] | USA | High-income |
| Sanchez, 2016[121] | Spain | High-income |
| Santos, 2017[122] | USA | High-income |
| Sapru, 2017[123] | USA | High-income |
| Saw, 2013[124] | USA | High-income |
| Schwingel, 2017[125] | USA | High-income |
| Shafer, 2017[126] | USA | High-income |
| Shanahan, 2014[127] | USA | High-income |
| Shanks, 2016[128] | USA | High-income |
| Shubert, 2011[129] | USA | High-income |
| Sin, 2017[130] | USA | High-income |
| Smedegaard, 2017[131] | Denmark | High-income |
| Storm, 2016[132] | USA | High-income |
| Sweet, 2014[133] | Canada | High-income |
| Takeuchi, 2017[134] | Tonga | Upper-middle income |
| Tapp, 2014[135] | USA | High-income |
| Thomas, 2014[136] | Sweden | High-income |
| Thomas, 2015[137] | Sweden | High-income |
| Thomas, 2016[138] | USA | High-income |
| Tjia, 2015[139] | USA | High-income |
| Toobert, 2012[140] | USA | High-income |
| Ulbricht, 2014[141] | Germany | High-income |
| Van Acker, 2011[142] | Belgium | High-income |
| Van Hoye, 2015[143] | Norway and France | High-income & high-income |
| Vick, 2013[144] | USA | High-income |
| Vidrine, 2013[145] | USA | High-income |
| Viester, 2014[146] | Netherlands | High-income |
| Vriend, 2015[147] | Netherlands | High-income |
| Wallace, 2016[148] | USA | High-income |
| Weiss, 2015[149] | USA | High-income |
| Wozniak, 2015^a^[150] | Canada | High-income |
| Wozniak, 2015^b^[151] | Canada | High-income |
| Wozniak, 2015^c^[152] | Canada | High-income |
| Wozniak, 2015^d^[153] | Canada | High-income |
| Wozniak, 2016[154] | Canada | High-income |
| Yank, 2013[155] | USA | High-income |
| Yeh, 2014[156] | Taiwan | High-income |
| Young, 2012[157] | USA | High-income |

* The classification of a country was based on the 2018 World Bank classification criteria^[158]^

References

**References**

1. Adams EJ, Chalkley AE, Esliger DW, Sherar LB. Evaluation of the implementation of a whole-workplace walking programme using the RE-AIM framework. *BMC Public Health.* 2017;17(1):466.

2. Aerts I, Cumps E, Verhagen E, Mathieu N, Van Schuerbeeck S, Meeusen R. A 3-month jump-landing training program: a feasibility study using the RE-AIM framework. *Journal of Athletic Training.* 2013;48(3):296-305.

3. Aittasalo M, Rinne M, Pasanen M, Kukkonen-Harjula K, Vasankari T. Promoting walking among office employees - evaluation of a randomized controlled intervention with pedometers and e-mail messages. *BMC Public Health.* 2012;12:403.

4. Allicock M, Johnson L-S, Leone L, et al. Promoting fruit and vegetable consumption among members of black churches, Michigan and North Carolina, 2008-2010. *Preventing Chronic Disease.* 2013;10:E33.

5. Altpeter M, Gwyther LP, Kennedy SR, Patterson TR, Derence K. From evidence to practice: using the RE-AIM framework to adapt the REACHII caregiver intervention to the community. *Dementia.* 2015;14(1):104-113.

6. Anderson KA, Weber KV. Auto Therapy: Using Automobiles as Vehicles for Reminiscence With Older Adults. *J Gerontol Soc Work.* 2015;58(5):469-483.

7. Anderson WG, Puntillo K, Cimino J, et al. Palliative Care Professional Development for Critical Care Nurses: A Multicenter Program. *Am J Crit Care.* 2017;26(5):361-371.

8. Anesetti-Rothermel A, Noerachmanto N, Horn K, Dino G. Beyond reach and effectiveness: evaluating the not-on-tobacco (N-o-T) program in West Virginia from 2000 to 2005. *Health Promot Pract.* 2012;13(4):506-514.

9. Arbour-Nicitopoulos KP, Tomasone JR, Latimer-Cheung AE, Martin Ginis KA. Get in motion: an evaluation of the reach and effectiveness of a physical activity telephone counseling service for Canadians living with spinal cord injury. *PM R.* 2014;6(12):1088-1096.

10. Arrossi S, Paolino M, Thouyaret L, Laudi R, Campanera A. Evaluation of scaling-up of HPV self-collection offered by community health workers at home visits to increase screening among socially vulnerable under-screened women in Jujuy Province, Argentina. *Implement Sci.* 2017;12(1):17.

11. Austin G, Bell T, Caperchione C, Mummery WK. Translating research to practice: using the RE-AIM framework to examine an evidence-based physical activity intervention in primary school settings. *Health Promot Pract.* 2011;12(6):932-941.

12. Baba CT, Oliveira IM, Silva AEF, et al. Evaluating the impact of a walking program in a disadvantaged area: using the RE-AIM framework by mixed methods. *BMC Public Health.* 2017;17(1):709.

13. Banfield M, McGorm K, Sargent G. Health promotion in schools: a multi-method evaluation of an Australian School Youth Health Nurse Program. *BMC Nurs.* 2015;14:21.

14. Belkora J, Volz S, Loth M, et al. Coaching patients in the use of decision and communication aids: RE-AIM evaluation of a patient support program. *BMC Health Serv Res.*2015;15:209.

15. Bhardwaja B, Carroll NM, Raebel MA, et al. Improving prescribing safety in patients with renal insufficiency in the ambulatory setting: the Drug Renal Alert Pharmacy (DRAP) program. *Pharmacotherapy.* 2011;31(4):346-356.

16. Bhojani U, Kolsteren P, Criel B, et al. Intervening in the local health system to improve diabetes care: lessons from a health service experiment in a poor urban neighborhood in India. *Global Health Action.* 2015;8:28762.

17. Boersma P, van Weert JCM, van Meijel B, Dröes R-M. Implementation of the Veder contact method in daily nursing home care for people with dementia: a process analysis according to the RE-AIM framework. *Journal of Clinical Nursing.* 2017;26(3-4):436-455.

18. Brace AM, Padilla HM, DeJoy DM, Wilson MG, Vandenberg RJ, Davis M. Applying RE-AIM to the evaluation of FUEL Your Life : a worksite translation of DPP. *Health Promot Pract.* 2015;16(1):28-35.

19. Brinkley A, McDermott H, Munir F. Team Sport in the Workplace? A RE-AIM Process Evaluation of 'Changing the Game'. *AIMS Public Health.* 2017;4(5):466-489.

20. Brown JC, Verhagen E, van Mechelen W, Lambert MI, Draper CE. Coaches’ and referees’ perceptions of the BokSmart injury prevention programme. *International Journal of Sports Science & Coaching.* 2016;11(5):637-647.

21. Broyles LM, Gordon AJ, Rodriguez KL, Hanusa BH, Kengor C, Kraemer KL. Evaluation of a pilot training program in alcohol screening, brief intervention, and referral to treatment for nurses in inpatient settings. *Journal of Addictions Nursing.* 2013;24(1):8-19.

22. Brunisholz KD, Kim J, Savitz LA, et al. A Formative Evaluation of a Diabetes Prevention Program Using the RE-AIM Framework in a Learning Health Care System, Utah, 2013-2015. *Preventing Chronic Disease.* 2017;14:E58.

23. Buis LR, Hirzel L, Turske SA, Des Jardins TR, Yarandi H, Bondurant P. Use of a text message program to raise type 2 diabetes risk awareness and promote health behavior change (part II): assessment of participants' perceptions on efficacy. *J Med Internet Res.* 2013;15(12):e282.

24. Bukhari A, Fredericks L, Wylie-Rosett J. Strategies to promote high school students' healthful food choices. *J Nutr Educ Behav.* 2011;43(5):414-418.

25. Burke SM, Shapiro S, Petrella RJ, et al. Using the RE-AIM framework to evaluate a community-based summer camp for children with obesity: a prospective feasibility study. *BMC Obes.* 2015;2:21.

26. Caperchione CM, Duncan M, Kolt GS, et al. Examining an Australian physical activity and nutrition intervention using RE-AIM. *Health Promot Int.* 2016;31(2):450-458.

27. Carlfjord S, Andersson A, Bendtsen P, Nilsen P, Lindberg M. Applying the RE-AIM framework to evaluate two implementation strategies used to introduce a tool for lifestyle intervention in Swedish primary health care. *Health Promot Int.* 2012;27(2):167-176.

28. Carlfjord S, Lindberg M, Andersson A. Sustained use of a tool for lifestyle intervention implemented in primary health care: a 2-year follow-up. *J Eval Clin Pract.* 2013;19(2):327-334.

29. Casey MM, Telford A, Mooney A, Harvey JT, Eime RM, Payne WR. Linking secondary school physical education with community sport and recreation for girls: a process evaluation. *BMC Public Health.* 2014;14:1039.

30. Chao MT, Abercrombie PD, Santana T, Duncan LG. Applying the RE-AIM Framework to Evaluate Integrative Medicine Group Visits Among Diverse Women with Chronic Pelvic Pain. *Pain Manag Nurs.* 2015;16(6):920-929.

31. Chen I, Money D, Yong P, Williams C, Allaire C. An Evaluation Model for a Multidisciplinary Chronic Pelvic Pain Clinic: Application of the RE-AIM Framework. *J Obstet Gynaecol Can.* 2015;37(9):804-809.

32. Christian D, Todd C, Hill R, et al. Active children through incentive vouchers - evaluation (ACTIVE): a mixed-method feasibility study. *BMC Public Health.* 2016;16:890.

33. Clemson L, Donaldson A, Hill K, Day L. Implementing person-environment approaches to prevent falls: a qualitative inquiry in applying the Westmead approach to occupational therapy home visits. *Aust Occup Ther J.* 2014;61(5):325-334.

34. Conlon BA, Kahan M, Martinez M, et al. Development and Evaluation of the Curriculum for BOLD (Bronx Oncology Living Daily) Healthy Living: a Diabetes Prevention and Control Program for Underserved Cancer Survivors. *J Cancer Educ.* 2015;30(3):535-545.

35. Conte KP, Odden MC, Linton NM, Harvey SM. Effectiveness of a Scaled-Up Arthritis Self-Management Program in Oregon: Walk With Ease. *Am J Public Health.* 2016;106(12):2227-2230.

36. Cook PF, Richardson G, Wilson A. Motivational interviewing training to promote Head Start children's adherence to oral health care recommendations: results of a program evaluation. *J Public Health Dent.* 2013;73(2):147-150.

37. Damschroder LJ, Reardon CM, AuYoung M, et al. Implementation findings from a hybrid III implementation-effectiveness trial of the Diabetes Prevention Program (DPP) in the Veterans Health Administration (VHA). *Implement Sci.* 2017;12(1):94.

38. Dubuy V, De Cocker K, De Bourdeaudhuij I, et al. Evaluation of a workplace intervention to promote commuter cycling: a RE-AIM analysis. *BMC Public Health.* 2013;13:587.

39. Duffy SA, Ewing LA, Louzon SA, Ronis DL, Jordan N, Harrod M. Evaluation and costs of volunteer telephone cessation follow-up counseling for Veteran smokers discharged from inpatient units: a quasi-experimental, mixed methods study. *Tob Induc Dis.* 2015;13(1):4.

40. Duffy SA, Ronis DL, Ewing LA, et al. Implementation of the Tobacco Tactics intervention versus usual care in Trinity Health community hospitals. *Implement Sci.* 2016;11(1):147.

41. Dunton GF, Liao Y, Grana R, et al. State-wide dissemination of a school-based nutrition education programme: a RE-AIM (Reach, Efficacy, Adoption, Implementation, Maintenance) analysis. *Public Health Nutr.* 2014;17(2):422-430.

42. Eisen JC, Marko-Holguin M, Fogel J, et al. Pilot Study of Implementation of an Internet-Based Depression Prevention Intervention (CATCH-IT) for Adolescents in 12 US Primary Care Practices: Clinical and Management/Organizational Behavioral Perspectives. *Prim Care Companion CNS Disord.* 2013;15(6).

43. Elliott T, Trevena H, Sacks G, et al. A systematic interim assessment of the Australian Government's Food and Health Dialogue. *Med J Aust.* 2014;200(2):92-95.

44. Estabrook B, Zapka J, Lemon SC. Evaluating the implementation of a hospital work-site obesity prevention intervention: applying the RE-AIM framework. *Health Promot Pract.* 2012;13(2):190-197.

45. Evenson KR, Brownson RC, Satinsky SB, Eyler AA, Kohl HW. The U.S. National Physical Activity Plan: dissemination and use by public health practitioners. *Am J Prev Med.* 2013;44(5):431-438.

46. Finocchario-Kessler S, Odera I, Okoth V, et al. Lessons learned from implementing the HIV infant tracking system (HITSystem): A web-based intervention to improve early infant diagnosis in Kenya. *Healthc (Amst).* 2015;3(4):190-195.

47. Folta SC, Seguin RA, Chui KKH, et al. National Dissemination of StrongWomen-Healthy Hearts: A Community-Based Program to Reduce Risk of Cardiovascular Disease Among Midlife and Older Women. *Am J Public Health.* 2015;105(12):2578-2585.

48. Folta SC, Lichtenstein AH, Seguin RA, et al. The StrongWomen-Healthy Hearts program in Pennsylvania: RE-AIM analysis. *Transl Behav Med.* 2015;5(1):94-102.

49. Fortney J, Enderle M, McDougall S, et al. Implementation outcomes of evidence-based quality improvement for depression in VA community based outpatient clinics. *Implement Sci.* 2012;7:30.

50. Fortney JC, Enderle MA, Clothier JL, Otero JM, Williams JS, Pyne JM. Population level effectiveness of implementing collaborative care management for depression. *Gen Hosp Psychiatry.* 2013;35(5):455-460.

51. Freyer-Adam J, Baumann S, Haberecht K, et al. In-person and computer-based alcohol interventions at general hospitals: reach and retention. *Eur J Public Health.* 2016;26(5):844-849.

52. Gainforth HL, Latimer-Cheung AE, Athanasopoulos P, Martin Ginis KA. Examining the feasibility and effectiveness of a community-based organization implementing an event-based knowledge mobilization initiative to promote physical activity guidelines for people with spinal cord injury among support personnel. *Health Promot Pract.* 2015;16(1):55-62.

53. Glasgow RE, Dickinson P, Fisher L, et al. Use of RE-AIM to develop a multi-media facilitation tool for the patient-centered medical home. *Implement Sci.* 2011;6:118.

54. Glasgow RE, Askew S, Purcell P, et al. Use of RE-AIM to Address Health Inequities: Application in a low-income community health center based weight loss and hypertension self-management program. *Transl Behav Med.* 2013;3(2):200-210.

55. Goode AD, Eakin EG. Dissemination of an evidence-based telephone-delivered lifestyle intervention: factors associated with successful implementation and evaluation. *Transl Behav Med.* 2013;3(4):351-356.

56. Gordon P, Camhi E, Hesse R, et al. Processes and outcomes of developing a continuity of care document for use as a personal health record by people living with HIV/AIDS in New York City. *Int J Med Inform.* 2012;81(10):e63-73.

57. Grow HMG, Hencz P, Verbovski MJ, et al. Partnering for success and sustainability in community-based child obesity intervention: seeking to help families ACT! *Fam Community Health.* 2014;37(1):45-59.

58. Hagedorn HJ, Stetler CB, Bangerter A, Noorbaloochi S, Stitzer ML, Kivlahan D. An implementation-focused process evaluation of an incentive intervention effectiveness trial in substance use disorders clinics at two Veterans Health Administration medical centers. *Addict Sci Clin Pract.* 2014;9:12.

59. Hanson LC, Green MA, Hayes M, et al. Circles of Care: Implementation and Evaluation of Support Teams for African Americans With Cancer. *Health Educ Behav.* 2014;41(3):291-298.

60. Harden SM, Fanning JT, Motl RW, McAuley E, Estabrooks PA. Determining the reach of a home-based physical activity program for older adults within the context of a randomized controlled trial. *Health Educ Res.* 2014;29(5):861-869.

61. Harden SM, Johnson SB, Almeida FA, Estabrooks PA. Improving physical activity program adoption using integrated research-practice partnerships: an effectiveness-implementation trial. *Transl Behav Med.* 2017;7(1):28-38.

62. Headley CM, Payne LL, Keller MJ. N'Balance: A Community-Based Fall-Prevention Intervention With Older Adults—Lessons Learned. *Activities, Adaptation & Aging.* 2013;37(1):47-62.

63. Huang S-K, Wang P-J, Tseng W-F, et al. NHI-PharmaCloud in Taiwan--A preliminary evaluation using the RE-AIM framework and lessons learned. *Int J Med Inform.* 2015;84(10):817-825.

64. Huang K-Y, Nakigudde J, Rhule D, et al. Transportability of an Evidence-Based Early Childhood Intervention in a Low-Income African Country: Results of a Cluster Randomized Controlled Study. *Prev Sci.* 2017;18(8):964-975.

65. Hussain T, Franz W, Brown E, et al. The Role of Care Management as a Population Health Intervention to Address Disparities and Control Hypertension: A Quasi-Experimental Observational Study. *Ethn Dis.* 2016;26(3):285-294.

66. Huye HF, Connell CL, Crook LB, Yadrick K, Zoellner J. Using the RE-AIM Framework in formative evaluation and program planning for a nutrition intervention in the Lower Mississippi Delta. *J Nutr Educ Behav.* 2014;46(1):34-42.

67. Hynes DM, Fischer MJ, Schiffer LA, et al. Evaluating a novel health system intervention for chronic kidney disease care using the RE-AIM framework: Insights after two years. *Contemp Clin Trials.* 2017;52:20-26.

68. Jaipakdee J, Jiamjarasrangsi W, Lohsoonthorn V, Lertmaharit S. Effectiveness of a self-management support program for Thais with type 2 diabetes: Evaluation according to the RE-AIM framework. *Nurs Health Sci.* 2015;17(3):362-369.

69. Jang Y, Yoon H, Marti CN, Kim MT. Aging IQ Intervention with Older Korean Americans: A Comparison of Internet-Based and In-Class Education. *Educational Gerontology.* 2015;41(9):642-652.

70. Janssen M, Toussaint HM, van Mechelen W, Verhagen EALM. Translating the PLAYgrounds program into practice: a process evaluation using the RE-AIM framework. *J Sci Med Sport.* 2013;16(3):211-216.

71. Jenkinson KA, Naughton G, Benson AC. The GLAMA (Girls! Lead! Achieve! Mentor! Activate!) physical activity and peer leadership intervention pilot project: a process evaluation using the RE-AIM framework. *BMC Public Health.* 2012;12:55.

72. Jenny GJ, Brauchli R, Inauen A, Füllemann D, Fridrich A, Bauer GF. Process and outcome evaluation of an organizational-level stress management intervention in Switzerland. *Health Promot Int.* 2015;30(3):573-585.

73. Jeong H-J, Jo H-S, Oh M-K, Oh H-W. Applying the RE-AIM Framework to Evaluate the Dissemination and Implementation of Clinical Practice Guidelines for Sexually Transmitted Infections. *J Korean Med Sci.* 2015;30(7):847-852.

74. Jones A, Magnusson R, Swinburn B, et al. Designing a Healthy Food Partnership: lessons from the Australian Food and Health Dialogue. *BMC Public Health.* 2016;16:651.

75. Kahwati LC, Lance TX, Jones KR, Kinsinger LS. RE-AIM evaluation of the Veterans Health Administration's MOVE! Weight Management Program. *Transl Behav Med.* 2011;1(4):551-560.

76. Kastirke N, John U, Goeze C, Sannemann J, Ulbricht S. Reaching families at their homes for an intervention to reduce tobacco smoke exposure among infants. *J Community Health.* 2013;38(2):215-220.

77. Kim AE, Towers A, Renaud J, et al. Application of the RE-AIM framework to evaluate the impact of a worksite-based financial incentive intervention for smoking cessation. *J Occup Environ Med.* 2012;54(5):610-614.

78. Kirchner JE, Ritchie MJ, Pitcock JA, Parker LE, Curran GM, Fortney JC. Outcomes of a partnered facilitation strategy to implement primary care-mental health. *J Gen Intern Med.* 2014;29 Suppl 4:904-912.

79. Koorts H, Gillison F. Mixed method evaluation of a community-based physical activity program using the RE-AIM framework: practical application in a real-world setting. *BMC Public Health.* 2015;15:1102.

80. Kozica SL, Lombard CB, Harrison CL, Teede HJ. Evaluation of a large healthy lifestyle program: informing program implementation and scale-up in the prevention of obesity. *Implement Sci.* 2016;11(1):151.

81. Krist AH, Phillips SM, Sabo RT, et al. Adoption, reach, implementation, and maintenance of a behavioral and mental health assessment in primary care. *Ann Fam Med.* 2014;12(6):525-533.

82. Lane A, Murphy N, Bauman A. An effort to 'leverage' the effect of participation in a mass event on physical activity. *Health Promot Int.* 2015;30(3):542-551.

83. Larsen AL, Robertson T, Dunton G. RE-AIM analysis of a randomized school-based nutrition intervention among fourth-grade classrooms in California. *Transl Behav Med.* 2015;5(3):315-326.

84. Larsen AL, Liao Y, Alberts J, Huh J, Robertson T, Dunton GF. RE-AIM Analysis of a School-Based Nutrition Education Intervention in Kindergarteners. *J Sch Health.* 2017;87(1):36-46.

85. Lee RE, Soltero EG, Ledoux TA, et al. Sustainability via Active Garden Education: Translating Policy to Practice in Early Care and Education. *J Sch Health.* 2019;89(4):257-266.

86. Leenaars KEF, Smit E, Wagemakers A, Molleman GRM, Koelen MA. Exploring the impact of the care sport connector in the Netherlands. *BMC Public Health.* 2017;17(1):813.

87. Leveille SG, Walker J, Ralston JD, Ross SE, Elmore JG, Delbanco T. Evaluating the impact of patients' online access to doctors' visit notes: designing and executing the OpenNotes project. *BMC Med Inform Decis Mak.* 2012;12:32.

88. Lewis ZH, Ottenbacher KJ, Fisher SR, et al. The feasibility and RE-AIM evaluation of the TAME health pilot study. *Int J Behav Nutr Phys Act.* 2017;14(1):106.

89. Li F, Harmer P, Stock R, et al. Implementing an evidence-based fall prevention program in an outpatient clinical setting. *J Am Geriatr Soc.* 2013;61(12):2142-2149.

90. Liddy C, Johnston S, Nash K, Irving H, Davidson R. Implementation and evolution of a regional chronic disease self-management program. *Can J Public Health.* 2016;107(2):e194-e201.

91. Lindblom H, Waldén M, Carlfjord S, Hägglund M. Implementation of a neuromuscular training programme in female adolescent football: 3-year follow-up study after a randomised controlled trial. *Br J Sports Med.* 2014;48(19):1425-1430.

92. Liu G, Perkins A. Using a lay cancer screening navigator to increase colorectal cancer screening rates. *J Am Board Fam Med.* 2015;28(2):280-282.

93. Lum HD, Jones J, Matlock DD, et al. Advance Care Planning Meets Group Medical Visits: The Feasibility of Promoting Conversations. *Ann Fam Med.* 2016;14(2):125-132.

94. Mahabee-Gittens EM, Dixon CA, Vaughn LM, Duma EM, Gordon JS. Parental tobacco screening and counseling in the pediatric emergency department: practitioners' attitudes, perceived barriers, and suggestions for implementation and maintenance. *J Emerg Nurs.* 2014;40(4):336-345.

95. Martínez-Donate AP, Riggall AJ, Meinen AM, et al. Evaluation of a pilot healthy eating intervention in restaurants and food stores of a rural community: a randomized community trial. *BMC Public Health.* 2015;15:136.

96. Maxwell AE, Danao LL, Cayetano RT, Crespi CM, Bastani R. Implementation of an evidence-based intervention to promote colorectal cancer screening in community organizations: a cluster randomized trial. *Transl Behav Med.* 2016;6(2):295-305.

97. Meyer C, Ulbricht S, Gross B, et al. Adoption, reach and effectiveness of computer-based, practitioner delivered and combined smoking interventions in general medical practices: a three-arm cluster randomized trial. *Drug Alcohol Depend.* 2012;121(1-2):124-132.

98. Mielenz TJ, Jia H, Seefeld E, et al. Translating using RE-AIM of a falls behavior change program among an assisted living population. *Fam Community Health.* 2014;37(2):147-154.

99. More NS, Das S, Bapat U, et al. Community resource centres to improve the health of women and children in informal settlements in Mumbai: a cluster-randomised, controlled trial. *Lancet Glob Health.* 2017;5(3):e335-e349.

100. Nigg C, Geller K, Adams P, Hamada M, Hwang P, Chung R. Successful dissemination of Fun 5 - a physical activity and nutrition program for children. *Transl Behav Med.* 2012;2(3):276-285.

101. Nothwehr F, Haines H, Chrisman M, Schultz U. Statewide dissemination of a rural, non-chain restaurant intervention: adoption, implementation and maintenance. *Health Educ Res.* 2014;29(3):433-441.

102. Odgers-Jewell K, Isenring E, Thomas R, Reidlinger DP. Process evaluation of a patient-centred, patient-directed, group-based education program for the management of type 2 diabetes mellitus. *Nutr Diet.* 2017;74(3):243-252.

103. Oldroyd JC, White S, Stephens M, Neil AA, Nanayakkara V. Program Evaluation of the Inner South Community Health Oral Health Program for Priority Populations. *J Health Care Poor Underserved.* 2017;28(3):1222-1239.

104. Olstad DL, Ball K, Abbott G, et al. A process evaluation of the Supermarket Healthy Eating for Life (SHELf) randomized controlled trial. *Int J Behav Nutr Phys Act.* 2016;13:27.

105. Paez DC, Reis RS, Parra DC, et al. Bridging the gap between research and practice: an assessment of external validity of community-based physical activity programs in Bogotá, Colombia, and Recife, Brazil. *Transl Behav Med.* 2015;5(1):1-11.

106. Palacio A, Keller VF, Chen J, Tamariz L, Carrasquillo O, Tanio C. Can Physicians Deliver Chronic Medications at the Point of Care? *Am J Med Qual.* 2016;31(3):256-264.

107. Paone D. Using RE-AIM to evaluate implementation of an evidence-based program: a case example from Minnesota. *J Gerontol Soc Work.* 2014;57(6-7):602-625.

108. Parahoo K, McKenna S, Prue G, McSorley O, McCaughan E. Facilitators' delivery of a psychosocial intervention in a controlled trial for men with prostate cancer and their partners: a process evaluation. *J Adv Nurs.* 2017;73(7):1620-1631.

109. Patel MR, Shah S, Cabana MD, et al. Translation of an evidence-based asthma intervention: Physician Asthma Care Education (PACE) in the United States and Australia. *Prim Care Respir J.* 2013;22(1):29-36.

110. Payne JM, France KE, Henley N, et al. RE-AIM evaluation of the Alcohol and Pregnancy Project: educational resources to inform health professionals about prenatal alcohol exposure and fetal alcohol spectrum disorder. *Eval Health Prof.* 2011;34(1):57-80.

111. Peels DA, Bolman C, Golsteijn RHJ, et al. Differences in reach and attrition between Web-based and print-delivered tailored interventions among adults over 50 years of age: clustered randomized trial. *J Med Internet Res.* 2012;14(6):e179.

112. Peralta CA, Frigaard M, Rubinsky AD, et al. Implementation of a pragmatic randomized trial of screening for chronic kidney disease to improve care among non-diabetic hypertensive veterans. *BMC Nephrol.* 2017;18(1):132.

113. Perry RA, Golley RK, Hartley J, Magarey AM. The adaptation and translation of the PEACH™ RCT intervention: the process and outcomes of the PEACH™ in the community trial. *Public Health.* 2017;153:154-162.

114. Poulos RG, Donaldson A. Is sports safety policy being translated into practice: what can be learnt from the Australian rugby union Mayday procedure? *Br J Sports Med.* 2012;46(8):585-590.

115. Poulos RG, Donaldson A. Improving the diffusion of safety initiatives in community sport. *J Sci Med Sport.* 2015;18(2):139-144.

116. Quinn E, Johnson DB, Krieger J, MacDougall E, Payne E, Chan NL. Developing local board of health guidelines to promote healthy food access - King County, Washington, 2010-2012. *Preventing Chronic Disease.* 2015;12:E58.

117. Rasmussen CDN, Larsen AK, Holtermann A, Søgaard K, Jørgensen MB. Adoption of workplaces and reach of employees for a multi-faceted intervention targeting low back pain among nurses' aides. *BMC Med Res Methodol.* 2014;14:60.

118. Resnick B, Galik E, Gruber-Baldini A, Zimmerman S. Understanding dissemination and implementation of a new intervention in assisted living settings: the case of function-focused care. *J Appl Gerontol.* 2013;32(3):280-301.

119. Resnick B, Galik E, Vigne E. Translation of function-focused care to assisted living facilities. *Fam Community Health.* 2014;37(2):155-165.

120. Samia LW, Aboueissa A-M, Halloran J, Hepburn K. The Maine Savvy Caregiver Project: translating an evidence-based dementia family caregiver program within the RE-AIM Framework. *J Gerontol Soc Work.* 2014;57(6-7):640-661.

121. Sánchez A, Silvestre C, Campo N, Grandes G, Pre DErg. Type-2 diabetes primary prevention program implemented in routine primary care: a process evaluation study. *Trials.* 2016;17(1):254.

122. Santos SLZ, Tagai EK, Scheirer MA, et al. Adoption, reach, and implementation of a cancer education intervention in African American churches. *Implement Sci.* 2017;12(1):36.

123. Sapru S, Berktold J, Crews JE, et al. Applying RE-AIM to evaluate two community-based programs designed to improve access to eye care for those at high-risk for glaucoma. *Eval Program Plann.* 2017;65:40-46.

124. Saw A, Kim J, Lim J, Powell C, Tong EK. Smoking cessation counseling for Asian immigrants with serious mental illness: using RE-AIM to understand challenges and lessons learned in primary care-behavioral health integration. *Health Promot Pract.* 2013;14(5 Suppl):70S-79S.

125. Schwingel A, Gálvez P, Linares D, Sebastião E. Using a Mixed-Methods RE-AIM Framework to Evaluate Community Health Programs for Older Latinas. *J Aging Health.* 2017;29(4):551-593.

126. Shafer E, Bergeron N, Smith-Ray R, Robson C, O'Koren R. A nationwide pharmacy chain responds to the opioid epidemic. *J Am Pharm Assoc (2003).* 2017;57(2S):S123-S129.

127. Shanahan M, Fleming P, Nocera M, Sullivan K, Murphy R, Zolotor A. Process evaluation of a statewide abusive head trauma prevention program. *Eval Program Plann.* 2014;47:18-25.

128. Shanks CB, Harden S. A Reach, Effectiveness, Adoption, Implementation, Maintenance Evaluation of Weekend Backpack Food Assistance Programs. *Am J Health Promot.* 2016;30(7):511-520.

129. Shubert TE, Altpeter M, Busby-Whitehead J. Using the RE-AIM framework to translate a research-based falls prevention intervention into a community-based program: lessons learned. *J Safety Res.* 2011;42(6):509-516.

130. Sin M-K, Yip M-P, Kimura A, Tu S-P. Facilitators and Hindrances of Implementing Colorectal Cancer Screening Intervention Among Vietnamese Americans. *Cancer Nurs.* 2017;40(3):E41-E47.

131. Smedegaard S, Brondeel R, Christiansen LB, Skovgaard T. What happened in the 'Move for Well-being in School': a process evaluation of a cluster randomized physical activity intervention using the RE-AIM framework. *Int J Behav Nutr Phys Act.* 2017;14(1):159.

132. Storm JF, LePrevost CE, Tutor-Marcom R, Cope WG. Adapting Certified Safe Farm to North Carolina Agriculture: An Implementation Study. *J Agromedicine.* 2016;21(3):269-283.

133. Sweet SN, Ginis KAM, Estabrooks PA, Latimer-Cheung AE. Operationalizing the RE-AIM framework to evaluate the impact of multi-sector partnerships. *Implement Sci.* 2014;9:74.

134. Takeuchi R, Kawamura K, Kawamura S, et al. Evaluation of the child oral health promotion 'MaliMali' Programme based on schools in the Kingdom of Tonga. *Int Dent J.* 2017;67(4):229-237.

135. Tapp H, Kuhn L, Alkhazraji T, et al. Adapting community based participatory research (CBPR) methods to the implementation of an asthma shared decision making intervention in ambulatory practices. *J Asthma.* 2014;51(4):380-390.

136. Thomas K, Krevers B, Bendtsen P. Long-term impact of a real-world coordinated lifestyle promotion initiative in primary care: a quasi-experimental cross-sectional study. *BMC Fam Pract.* 2014;15:201.

137. Thomas K, Krevers B, Bendtsen P. Implementing healthy lifestyle promotion in primary care: a quasi-experimental cross-sectional study evaluating a team initiative. *BMC health services research.* 2015;15:31.

138. Thomas LN, Hill TF, Gaines A, Dollahite JS. Implementing Smarter Lunchrooms Makeovers in New York state middle schools: an initial process evaluation. *Arch Public Health.* 2016;74:41.

139. Tjia J, Field T, Mazor K, et al. Dissemination of Evidence-Based Antipsychotic Prescribing Guidelines to Nursing Homes: A Cluster Randomized Trial. *J Am Geriatr Soc.* 2015;63(7):1289-1298.

140. Toobert DJ, Glasgow RE, Strycker LA, Barrera M, King DK. Adapting and RE-AIMing a heart disease prevention program for older women with diabetes. *Transl Behav Med.* 2012;2(2):180-187.

141. Ulbricht S, Groß S, Meyer C, Hannöver W, Nauck M, John U. Reducing tobacco smoke exposure in children aged below 4 years - a randomized controlled trial. *Prev Med.* 2014;69:208-213.

142. Van Acker R, De Bourdeaudhuij I, De Cocker K, Klesges LM, Cardon G. The impact of disseminating the whole-community project '10,000 Steps': a RE-AIM analysis. *BMC Public Health.* 2011;11:3.

143. Van Hoye A, Larsen T, Sovik M, et al. Evaluation of the Coaches Educators training implementation of the PAPA project: A comparison between Norway and France. *Scand J Med Sci Sports.* 2015;25(5):e539-546.

144. Vick L, Duffy SA, Ewing LA, Rugen K, Zak C. Implementation of an inpatient smoking cessation programme in a Veterans Affairs facility. *Journal of Clinical Nursing.* 2013;22(5-6):866-880.

145. Vidrine JI, Shete S, Cao Y, et al. Ask-Advise-Connect: a new approach to smoking treatment delivery in health care settings. *JAMA Intern Med.* 2013;173(6):458-464.

146. Viester L, Verhagen EALM, Bongers PM, van der Beek AJ. Process evaluation of a multifaceted health program aiming to improve physical activity levels and dietary patterns among construction workers. *J Occup Environ Med.* 2014;56(11):1210-1217.

147. Vriend I, Coehoorn I, Verhagen E. Implementation of an app-based neuromuscular training programme to prevent ankle sprains: a process evaluation using the RE-AIM Framework. *Br J Sports Med.* 2015;49(7):484-488.

148. Wallace AS, Perkhounkova Y, Sussman AL, Hein M, Chung SJ, Tripp-Reimer T. Implementation of collaborative goal setting for diabetes in community primary care. *Transl Behav Med.* 2016;6(2):202-211.

149. Weiss SM, Tobin JN, Lopez M, Simons H, Cook R, Jones DL. Translating an Evidence-Based Behavioral Intervention for Women Living with HIV into Clinical Practice: The SMART/EST Women's Program. *Int J Behav Med.* 2015;22(3):415-424.

150. Wozniak L, Soprovich A, Rees S, Johnson ST, Majumdar SR, Johnson JA. Challenges in Identifying Patients with Type 2 Diabetes for Quality-Improvement Interventions in Primary Care Settings and the Importance of Valid Disease Registries. *Can J Diabetes.* 2015;39 Suppl 3:S77-82.

151. Wozniak L, Soprovich A, Rees S, et al. Impact of Organizational Stability on Adoption of Quality-Improvement Interventions for Diabetes in Primary Care Settings. *Can J Diabetes.* 2015;39 Suppl 3:S100-112.

152. Wozniak L, Soprovich A, Rees S, Al Sayah F, Majumdar SR, Johnson JA. Contextualizing the Effectiveness of a Collaborative Care Model for Primary Care Patients with Diabetes and Depression (Teamcare): A Qualitative Assessment Using RE-AIM. *Can J Diabetes.* 2015;39 Suppl 3:S83-91.

153. Wozniak L, Soprovich A, Mundt C, Johnson JA, Johnson ST. Contextualizing the Proven Effectiveness of a Lifestyle Intervention for Type 2 Diabetes in Primary Care: A Qualitative Assessment Based on the RE-AIM Framework. *Can J Diabetes.* 2015;39 Suppl 3:S92-99.

154. Wozniak LA, Soprovich A, Rees S, Johnson ST, Majumdar SR, Johnson JA. A qualitative study examining healthcare managers and providers' perspectives on participating in primary care implementation research. *BMC health services research.* 2016;16:316.

155. Yank V, Stafford RS, Rosas LG, Ma J. Baseline reach and adoption characteristics in a randomized controlled trial of two weight loss interventions translated into primary care: a structured report of real-world applicability. *Contemp Clin Trials.* 2013;34(1):126-135.

156. Yeh Y-P, Chang C-J, Hsieh M-L, Wu H-T. Overcoming disparities in diabetes care: eight years' experience changing the diabetes care system in Changhua, Taiwan. *Diabetes Res Clin Pract.* 2014;106 Suppl 2:S314-322.

157. Young J, Gilwee J, Holman M, Messier R, Kelly M, Kessler R. Mental health, substance abuse, and health behavior intervention as part of the patient-centered medical home: a case study. *Transl Behav Med.* 2012;2(3):345-354.

158. The World Bank Country and Lending Groups Country Classification. 2018 Available from: https://datahelpdesk.worldbank.org/knowledgebase/articles/906519-world-bank-country-and-lending-groups. Accessed 1st March 2021.
